# Rheological Properties of Polyethylene Color Masterbatches Containing Pigment RED 122 (2,9-Dimethylquinacridone) Modified by Silanes Using Pulverization Method

**DOI:** 10.3390/polym17050618

**Published:** 2025-02-25

**Authors:** Magdalena Kozłowska, Magdalena Lipińska, Michał Okraska

**Affiliations:** Institute of Polymer and Dye Technology, Lodz University of Technology, 90-537 Lodz, Poland; magdalena.stefaniak@dokt.p.lodz.pl (M.K.); michal.okraska@p.lodz.pl (M.O.)

**Keywords:** polyethylene masterbatches, quinacridone pigments, viscoelastic properties

## Abstract

Polyethylene color masterbatches containing pigment RED 122, 2,9-dimethylquinacridone, ((2,9-dimethyl-5,12-dihydroquinolino[2,3-b]acridine-7,14-dione) modified by the pulverization method in ball mills were obtained. As pigment-modifying agents, isobutyltrimethoxysilane IBTMS and octyltrietoxysilane OTES were used. The viscoelastic properties of the prepared masterbatches were investigated by using an oscillation rotational rheometer. The impact of the 2 wt.% of coloring masterbatch on the rheological behavior of polyethylene during processing at 170 °C was analyzed. Storage shear modulus G′, loss shear modulus G″, complex viscosity η* and loss factor tan δ were analyzed. Modification prevents the agglomeration of modified pigment particles in the masterbatch, leading to a significant increase in the storage shear modulus G′, from 13.83 kPa (masterbatch containing pigment RED 122) to 58.74 kPa (pigment modified with 2 wt.% of IBTMS) and 49.67 kPa (pigment modified with 2 wt.% of OTES). The analysis of the continuous relaxation models showed that the modified pigment influenced the relaxation of melted polyethylene. The tendency of the silane-modified pigment to create its “own structure” in the polyethylene carrier via particle–particle interactions was estimated based on rotational tests at low and high shear rates. The larger area of viscosity loops was determined at 170 °C for the masterbatch containing 1 wt.% of OTES-modified pigment, 2574.44 Pas(1/s), as compared with the reference masterbatch, 464.88 Pas(1/s). The Carreau and Carreau–Yasuda viscosity models were applied to analyze the flow curve and the changes in viscosity as a function of the shear rate. After pigment modification, the zero shear viscosity µ_0_ of the mixtures of polyethylene/pigment masterbatch changed from 234.9 Pas (pigment RED 122) to 305.9 Pas (pigment modified with 1 wt.% of IBTMS). The influence of the modified pigments on the crystallization of polyethylene and its thermal stability was investigated. The temperatures of melting T_m_ were determined.

## 1. Introduction

Silanes belong to the group of hybrid organic–inorganic compounds. The general formula of alkoxysilanes is as follows:

R_n_Si(OX)_4−n_

where R—the group providing the organic capacity may be an alkyl group, an aromatic group, an organofunctional group, or combinations thereof; X—methoxy or ethoxy groups (generally alkoxy groups) capable of reacting with various forms of hydroxyl groups, releasing methanol or ethanol. Owing to these groups, it is possible to combine with inorganic substrates, filler or pigment [1]. Organosilanes are silanes that contain at least one Si–C bond (Si–CH_3_), synthesized from silica by carrying out several chemical reactions [2,3]. Silanes are divided into mono- and bifunctional ones due to their ability to interact with the polymer. Monofunctional silanes are designed to reduce surface polarity in order to reduce filler agglomeration. In turn, in bifunctional silanes, one of the functional groups is responsible for coupling the silica surface with a hydrophilic silanol, while the other group binds the hydrophobic polymer matrix [4]. Functional silanes are able to react with polymers to form chemical bonds and interpenetrate polymer networks (IPNs), and can also react with inorganic surfaces to form covalent metallo-siloxane bonds, which results in very good adhesion between silanes and the inorganic substrate. They therefore act as adhesion promoters [5]. There are also non-functional silanes that contain only reactive alkoxy groups (–OR), which are hydrolyzed to silanol groups and then react with hydroxyl groups on the surface of inorganic substrates [6]. Silanes have found applications in areas such as coatings [7], particles [8,9], composites [10,11], tire design [12], in construction [13], and dentistry [14].

Quinacridone, 5,12-dihydroquinolino(2,3-b)acridine-7-14-dione is an often-used pigment with a red-violet hue. It occurs in several polymorphic forms, which determine the properties of the pigment, including its shade. These polymorphs are insoluble or difficult to dissolve in water and other organic solvents [15]. The quinacridone pigment is characterized by a pentacyclic aromatic structure that enables absorption in the visible range. Unfortunately, this pigment belongs to organic compounds that exhibit aggregation problems [16]. The N–H and C=O groups present in the structure cause the formation of a network of hydrogen bonds, which affects its semiconducting properties [17]. The thermal stability of this pigment is above 500 °C [18]. It is used in thin-film transistors [19], semiconductors [20], organic solar cells [21] and in polymer dyeing [18]. In addition, quinacridone acts as a nucleating agent, which is due to its ability to form fine, dispersed crystals with high thermal stability.

The main problem with the application of quinacridone in polymer dyeing is the agglomeration of pigment particles, leading to inhomogeneous dispersion in polymer matrix. In the process of industrial applications, easy agglomeration of the pigment, uneven particles size and distribution of pigment particles in polymeric matrix can result in poor color quality of the colored product. Thus, for organic pigment, it is crucial to control the size of primary and secondary particles because this affects the pigment’s final color and opacity [22]. The pulverization method can be used to adjust the size of the primary particles. Various methods were applied to suppress the aggregation of primary particles and to improve the dispersion of pigments. Among them, the addition of dispersant [23,24], the grafting of polymers on the surface of pigment particles [25], and the encapsulation of pigments in polymers [26,27] were reported.

Yasuko Saito and coworkers proposed a method to improve the dispersion of quinacridone by using cellulose nanofibers [16] and chitosan nanofibers [28] as dispersants. Zeng H. et al. [23] used dispersants with various anchoring groups to disperse quinacridone pigment RED 122. Dispersants with the pyridine group exhibited better dispersion stability compared to those with a phenyl ring, an aminic function, and an alkyl chain. The interaction force between pigment and dispersants enhanced the stability, particle size, and size distribution of a pigment suspension. A promising solution is the elimination of the hydrogen-bonding network between the carbonyl groups and amine hydrogens in quinacridone through alkylation and synthesis of dialkyl (butyl, octyl, dodecyl) quinacridone derivatives [29]. Nevertheless, preventing pigment aggregation remains a challenge. Also, dispersing agents often cause a decline in the mechanical properties of colored plastics.

Here, we propose the modification of quinacridone particles using silanes with alkyl groups. The industry-acceptable pulverization method using ball mill (Pulverisette 5, Fritsch-GmBH, Idar-Oberstein, Germany) was applied to obtain modified pigments. Ball milling is a green and sustainable technique to grind powders into fine particles [30]. The pulverization method in a planetary ball mill uses shear and centrifugal forces and friction between steel grinding balls, a vessel and the powder being ground. The mechanical energy generated during grinding enables the physical decomposition of coarse particles into finer ones, which leads to an increase in the specific surface area of the particles. This allows for further optimization of the state of particle dispersion in composites [31,32]. Grinding parameters such as time, mixing speed, number of mixing cycles and the number of dispersing balls used determine the degree of powder grinding. A definite advantage of pulverization is its simplicity and low cost of filler modification. The grinding process in ball mills does not require the use of organic solvents; therefore, it is considered a green, environmentally friendly technology [33].

The silane-modified quinacridone pigments were used as a coloring additive to linear low-density polyethylene copolymer LLDPE. Various types of polyethylene (PE) are widely used in the packaging industry. According to the side chain branching, there are several types of PE: high-density (HDPE), low-density (LDPE), and linear low-density (LLDPE) [34]. Linear low-density polyethylene LLDPE [34] is a copolymer of ethylene and another longer olefin that is introduced to improve properties such as tensile strength or resistance to harsh environments. One of the four α-olefins (1-butene, 1-hexene, 4-methyl-1-pentene, and 1-octene) is usually polymerized with ethylene to form LLDPE. The amount of α-olefin is usually low compared to the amount of ethylene. Linear low-density polyethylene (LLDPE) is used in pipes, wires, and cables due to its high chemical stability, stress-cracking resistance and thermal properties [35,36]. The attractiveness of various polyethylene products for clients is often related to its color. The coloring substances, pigments, can be introduced into polymer melts. An interesting method that allows us to avoid pigment dusting on production lines is the use of highly concentrated pigment masterbatches, also called color concentrates. A color masterbatch is a mixture of pigments/dyes or other additives such as antioxidants, plasticizers, etc. previously incorporated into polymeric carrier during extrusion, and after cooling of the extruded ribbon, the material is cut in form of pellets [37]. Ultramarine blue pigment masterbatch was used to obtain the desired color quality of two types of polypropylene copolymer (PPCP) with varied melt flow rates and acrylonitrile butadiene styrene (ABS) copolymer [38]. A highly concentrated (30 wt.%) chitosan nanowhisker (CSW) masterbatch was blended with Nylon 6,10 to achieve high dispersion efficiency [39]. Eco-friendly natural pigments (spirulina, curcumin, beetroot and chlorophyllin) masterbatches were added at 2, 4 and 6 wt.%, as coloring additives during injection molding to poly(lactic acid) (PLA) and polybutylene succinate (PBS) [40]. The quinacridone pigments, ultramarine blue, and diketopyrrolopyrole masterbatches were used to dye polypropylene [41].

The masterbatch technology gives the plastic industry a number of advantages. Important here are the reduction in material cost as compared to dyeing in mass, simplifying the coloring process, and the reduction or complete elimination of inhalation of dust. The biggest challenge is the achievement of uniform homogenization of masterbatch components. Therefore, it is crucial to prevent pigment aggregation. The polarity and the surface free energy of quinacridone differs from that of polyethylene, generating interfacial tension during mixing in melt [42]. An element of novelty, not encountered in the literature to date, is the modification of quinacridone pigment using silanes. The aim of this research was to improve the miscibility of the pigment with the polymer matrix after its introduction to colored concentrates based on polyethylene. Modification with silanes was carried out to reduce the polarity of the Pigment RED 122 surface, and consequently to have a positive effect on its homogenization with non-polar polyethylene. Modification by the pulverization method, apart from unifying the pigment with silanes, enabled the fragmentation of aggregates present in the pigment, which additionally facilitated its homogenization with the polymer. Furthermore, it can influence the dispersion of the pigment in polymeric carrier. In our work, quinacridone was modified using two selected silane compounds, octyltriethoxysilane OTES and isobutyltrimethoxysilane IBTMS.

The concentrated masterbatches containing modified pigments were obtained. The rheological properties of LLDPE masterbatches were investigated by using an oscillation rotational rheometer Ares G2 (TA Instruments, New Castel, DE, USA). Rotational oscillation rheometers are a useful tool to study viscoelastic properties, including storage shear modulus G′, loss shear modulus G″, and the complex viscosity η* or shear viscosity μ of melted thermoplastic materials such as polyethylene’s [43,44,45]. Dynamic frequency sweep tests can provide vital information about the molecular structure, including dispersity and short/long branching of polymeric chains [43,45]. Industrial processes such as injection molding, bottle blow molding, sheet forming, fiber spinning, cable jacket extrusion, and rheological measurements correlate molecular weight, molecular weight distribution and branching of the molecular structure with such processing behavior as flow rate, die swell, and melt stability [45]. Thus, rheological tests can help to solve processability issues correlated with the properties of melt [45,46]. The dynamic oscillatory shear test can be used to convert the data of storage shear modulus G′ and loss shear modulus G″ into relaxation modulus G (t) and to analyze the differences in the relaxation behavior of various structure polyethylene [44]. Moreover, the rheological test can be performed continuously as the polymer undergoes the changes induced by temperature to determine the melting temperature T_m_ or the glass transition temperature T_g_ [45].

As a result of the improved dispersion of PR 122 in masterbatches, we expected an improvement in their rheological and processing properties. Additionally, the impact of the addition of 2 wt.% of the masterbatch on the viscoelastic properties of polyethylene was estimated. The influence of the incorporated masterbatch into the polyethylene on the relaxation behavior was analyzed. The effect of modified pigments on the crystallization of LLDPE and thermal properties was estimated.

## 2. Materials and Methods

### 2.1. Preparation of Modified Pigment and Colored Masterbatches

To obtain silane-modified pigments, the following were used:

1. C.I. Pigment RED 122, 2-9-dimethylquinacridone (2,9-dimethyl-5,12-dihydroquinolino [2,3-b]acridine-7,14-dione), CAS No. 980-26-7. Producent Zeya Chemicals BV. (Kerkenbos Nijmegen, The Netherlands);
2. Isobutyltrimethoxylsilane (trimethoxy(2-methylpropyl)silane), CAS No. 18395-30-7, producent Sigma-Aldrich (Poznań, Poland);
3. Triethoxy(octyl)silane (octyltriethoxysilane), CAS No. 2943-75-1, producent Sigma-Aldrich (Poznań, Poland).

**Figure 1:**
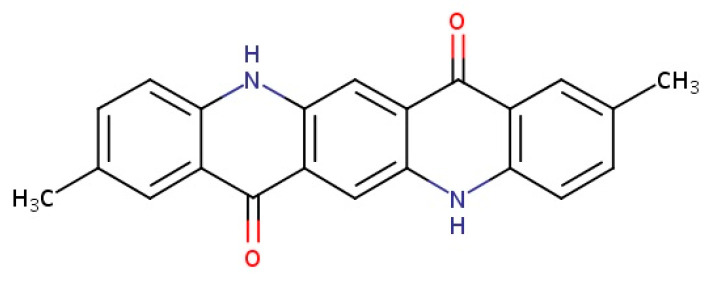
The chemical structure of 2-9-dimethylquinacridone pigment.

**Figure 2:**
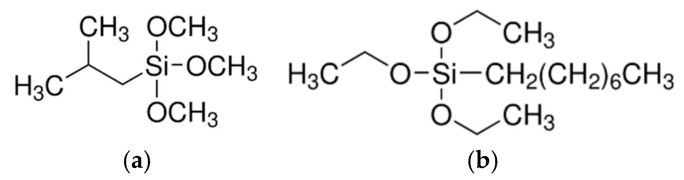
The chemical structure of modifying agents: (**a**) isobutyltrimethoxysilane IBTMS, (**b**) octyltriethoxysilane OTES.

The chemical structures of the compounds used are showed in Figure 1 and Figure 2.

The PM 200 planetary-ball mill (Pulverisette 5, Fritsch-GmBH, Idar-Oberstein, Germany) was used to modify the surface of the pigment. For this purpose, first, 100 g of the pigment was mixed with 1 or 2 wt.% of IBTMS or OTES silane and then the mixture was placed in a steel vessel with grinding balls. The 60 rpm rotational speed was set for 10 min. of grinding. The modified pigments are denoted as RED-1% IBTMS, RED-2% IBTMS, RED-1% OTES, RED-2% OTES.

**Figure 3:**
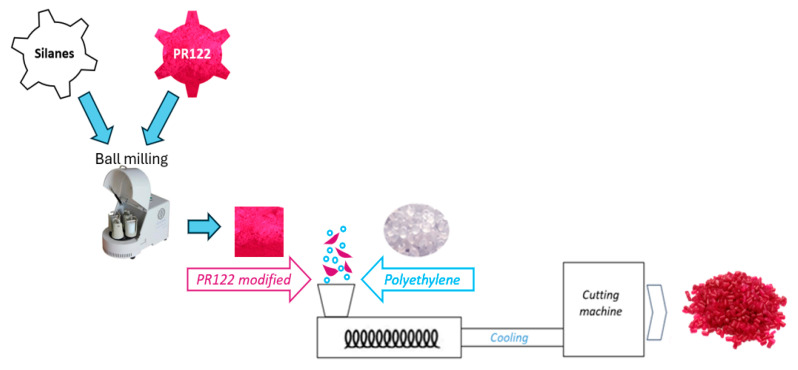
A diagram illustrating the method of pigment RED 122 modification by silanes used and the masterbatch production process.

For industrial reasons, surface modification of solid pigments using ball mills is an advantageous “green technology” due to low cost and the absence of solvents as compared with other methods, e.g., modification from solution. During the mixing of the pigment with the modifying substance, both deagglomeration, the physical breakdown of coarse particles into finer ones, and attachment of silane to the surface of the pigment RED 122 can occur. Thus, we selected the optimized pulverization method using a ball mill as an easy-to-use, fast, profitable and more ecological method of pigment modification. Furthermore, the modified pigments were added to the polymeric carrier and the colored masterbatches were prepared. The process of pigment modification and the masterbatch production is illustrated in Figure 3.

Linear low-density polyethylene LLDPE M50026 (Sabic Europe, Geelen, The Netherlands), denoted as PE, was used as a polymeric carrier in masterbatch formulation. SABIC^®^ LLDPE M500026 is a high-flow linear low-density polyethylene copolymer grade with a narrow molecular weight distribution dedicated to masterbatch compounding.

The parameters of LLDPE were as follows: melt flow rate, MFR, of 50 g·min^−1^ at 190 °C and 2.16 kg (ASTM D 1238 [47] and a density of 926 kg·m^−3^ [48]).

The C.I RED 122 and silane-modified pigments were used as a coloring additives to the LLDPE. The production of color concentrates took place in several stages (Figure 3). It began in the plasticizing system using a twin-screw extruder, where the components were dosed, melted, mixed with the supply of heat energy, and homogenized. The processing temperature was 170 °C, and the concentration of pigments in PE carrier was 30 wt.%.

The ribbon leaving the extruder head was cooled, and the last stage was the cutting of the obtained ribbon into uniform granules using a granulator. The prepared masterbatch containing pigment C.I. RED 122 is denoted as MB. Masterbatches containing silane-modified pigments are denoted as MB_1% OTES, MB_2% OTES, MB_1% IBTMS, MB_2% IBTMS.

Additionally, flat samples containing of 2 wt.% of masterbatch added to polyethylene during injection molding at 170 °C were obtained to study the influence of the masterbatch on the viscoelastic properties of polyethylene. The polyethylene samples containing 2 wt.% of various coloring concentrates are denoted as MB_1% IBTMS, 2 wt.% in PE; MB_2% IBTMS, 2 wt.% in PE; MB_1% OTES, 2 wt.% in PE; MB_2% OTES, 2 wt.% in PE. 

### 2.2. Characterization of Modified Pigments

#### 2.2.1. Aggregate Size Analysis Using DLS Method

The diameters of the formed aggregates of pigment were estimated based on the DLS dynamic light-scattering technique by using a Zetasizer Nano Serie S90 (Malvern Panalytical Ltd., Malvern, UK). The diameters of the aggregates were measured for water dispersions; the concentration was 5 g of pigment per 100 mL of distilled water. Additionally, the measurements were performed in paraffin oil (producent, PHU Olmax S.J., Lodz, Poland) to estimate the tendency to aggregation in non-polar medium. Before the measurements, the dispersions were stabilized, and an ultrasonic treatment in an ultrasonic bath (Bandelin Sonorex DT 255, Bandelin GmbH, Berlin, Germany) was applied for 15 min.

#### 2.2.2. The Surface Energy of Modified Pigments

The Owens–Wendt–Rabel–Kaelble (OWRK) method [42,49] is the standard procedure for calculating the surface free energy of solids, and it was used to estimate the effect of silane modification on the polar γ_p_ and dispersive γ_d_ components of the surface free energy γ of modified pigments. For this purpose, the tested pigments were wetted with several liquids (chloroform, 1,4-dioxane, ethyl alcohol, formamide). Measurements were performed at ambient temperatures using a tensiometer K100 MKII (Krüss GmbH, Hamburg, Germany). For each testing liquid, at least three measurements were taken. Additionally, the polarity parameter P was calculated based on Equation (1) [49]:

(1) $$P=\frac{\gamma_{p}}{\gamma }$$

where γ_p_—polar component of surface free energy; γ—total free surface energy.

#### 2.2.3. CIELab Measurement

In order to determine the influence of silane modification on the color profile of pigment, the CIE Lab measurement was performed using a Konica Minolta CM-36dG apparatus (Konica Minolta Inc. Japan, Chyoda-Tokio, Japan). A 4 mm diameter aperture was used. The CIE Lab model [50] was used, in which the two coordinates, a, b, describe the color profile. Positive values of a coordinate determine the shade of red color, and negative values of a coordinate refer to the shade of green color. Furthermore, the positive values of the b coordinate refer to the shade of yellow and the negative values of the b coordinate determine the shade of blue color. Parameter L is the lightness of color with the scale in a range from 0 (black) to 100 (white). The difference in color between two samples is calculated as the ΔE parameter according to Equation (2) [50]:

(2) $$∆E=\sqrt{(∆L{)}^{2}+(∆a{)}^{2}+(∆b{)}^{2}}$$

where L—lightness of color; a—red/green coordinate; b—yellow/blue coordinate.

The measurement of color was carried out for the pigment pressed in the form of pills, and the diameter of the pills was 10 mm. Three pills of each pigment were made, each measured three times. The results obtained were averaged.

### 2.3. Analysis of Viscoelastic Properties of Melted Masterbatches at 170 °C

Dynamic rheological measurements were carried out using an oscillation rotational rheometer ARES G2 (TA Instruments, New Castel, DE, USA), and plate–plate geometry with a diameter of 25 mm was used during all tests. The dynamic viscoelastic measurements were made based on (1) an amplitude sweep test in which the oscillation strain was programed to change in the range of 0.1–5% and the angular frequency was constant 10 rad·s^−1^; (2) a frequency sweep test in which the frequency was programmed to change in the range of 0.1–628 rad·s^−1^ under constant oscillations strain 0.5%; (3) a temperature sweep test in which the temperature was programmed to change in the range of 110–220 °C, the heating rate was 5 °C·min^−1^, and constant oscillation strain 0.02% and angular frequency 10 rad·s^−1^ were applied. The storage shear modulus G′, loss shear modulus G″, loss factor tan δ, complex viscosity η* were determined and analyzed.

The relaxation data and the continuous relaxation spectrum H(lnτ) were obtained by fitting oscillation data measured as a function of frequency in the range of 0.1–628 rad·s^−1^. Numerical fitting of the determined values of storage shear modulus G′ and loss shear modulus G″ was carried out by using the TRIOS^®^ Software (Trios v3 1.5.3696, TA Instruments, New Castel, DE, USA). The spectrum was discretized using 100 steps and represents all the pair-fitted [H_i_, τ_i_] parameters. The level of correlation was R^2^ = 0.998–0.999.

### 2.4. Analysis of Shear Viscosity µ and Flow of Melted Masterbatches at 170 °C

Rotational rheological measurements were carried out using an oscillation rotational rheometer ARES G2 (TA Instruments, New Castel, DE, USA), and plate–plate geometry with a diameter of 25 mm was used during all tests. The performed rotational measurements were as follows: (1) Steady flow sweep: shear viscosity µ was determined as a function of shear rate $\dot{\gamma }$. The applied shear rate was 0.001–300 s^−1^. The temperature of the test was 170 °C. Before the measurement, melted samples were conditioned at 170 °C during 60 s. (2) Hysteresis loop of shear viscosity: the measurement was carried out at 170 °C using two steps; in the first step, the applied shear rate increased in the range of 0.01–20 s^−1^ for 60 s; in the second step, the applied shear rate decreased in the range of 20–0.01 s^−1^. The area under the viscosity plots was determined and the area of the hysteresis loop was calculated.

The numerical fitting of the determined values of shear viscosity to calculate the zero shear viscosity μ_0_, the infinite viscosity μ_∞_, the consistency k (characteristic time), the power law index n, and a parameter describing the transition between a Newtonian plateau and the power law region was carried out by using the TRIOS^®^ Software (Trios v3 1.5.3696, TA Instruments, New Castel, DE, USA). The level of correlation was R^2^ = 0.996–0.999.

### 2.5. The DSC and TGA Analysis

A DSC1 apparatus (Mettler Toledo, Ithaca, NY, USA) was used. The samples were tested under a nitrogen atmosphere. Three heating/cooling/heating steps from −100 to 200 °C were performed; the heating rate was 10 °C·min^−1^. After the first heating rate, the isothermal step was performed for 1 min at 200 °C to eliminate the thermal history of the sample. The melting temperature T_m_, the enthalpy of melting ΔH_m_, the temperature of crystallization T_c_, and the enthalpy of crystallization ΔH_c_ were determined for every sample of PE containing 2% of colored masterbatches; the degree of crystallinity was calculated based on the following Equation (3) [51]:

(3) $$\chi_{c}=\frac{\Delta H_{m}}{\Delta H_{m}^{0}*w_{PE}}\cdot 100\%$$

where ΔH_m_ is the enthalpy of melting, and ΔH_m_^0^ is the theoretical enthalpy of melting of 100% crystalline polyethylene ΔH_m_^0^ = 293 J·g^−1^ [51,52]; w_PE_—polyethylene content in sample; and w_PE_—is the weight fraction of polyethylene.

A TGA/DSC1 (Mettler Toledo, Ithaca, NY, USA) apparatus was used to study the thermal properties and the weight loss of the sample during heating. During measurements, the samples of polyethylenes were heated from 25 to 600 °C under an argon atmosphere and from 600 to 900 °C in air; the heating rate was 10 °C·min^−1^. The TGA measurements for pigments were performed under an argon atmosphere.

### 2.6. SEM Analysis

The tendency to aggregate was estimated based on the SEM picture analysis of the pigment and modified pigment powders using a LEO 1530 Gemini SEM Microscope (Zeiss/Leo, Oberkochen, Germany) scanning electron microscope. Additionally, the examination of the cryogenic fracture surfaces of masterbatches and the polyethylene samples containing 2 wt.% of masterbatch was carried out. In these investigations, samples with a graphite-coated structure were used.

## 3. Results

### 3.1. The Influence of Modification on the Tendency Toward Agglomeration, Surface Energy of Pigment and Thermal Properties of Pigment RED 122

The tendency toward agglomeration was estimated, based on the DLS measurements, for the dispersion of pigment in two solvents, water (polar environment) and paraffine oil (hydrophobic environment), the low-molecular model of polyethylene. The range of the determined diameter of the formed pigment aggregates before and after modification is compiled in Table 1 and Table 2. In the Supplementary Materials (Figures S1–S4), DLS plots for studied dispersions of modified pigments in water and paraffine oil are compiled, showing the number percent as a function of size.

The modification of pigment surface by selected IBTMS and OTES silanes reduced the range of the formed aggregates in both polar medium water and non-polar medium paraffin oil (Table 1 and Table 2). In water, the aggregate range decreased below 1000 nm compared to the unmodified pigment. A stronger decrease in the aggregate range is observed for the pigment modified with OTES. In this case, the lower size limit is about half that of RED 122 and reaches values below 100 nm. The size of the main fraction was also lower for RED-OTES and its content in the main fraction increased. The measurement of dispersion in a nonpolar medium brought the same conclusions as in the case of the measurement in water. Again, a decrease in the size range of aggregates was observed for the silane-modified pigment, and the lowest was shown by the pigment modified with OTES. It is worth noting that the size of the main fraction of the modified pigment was more than twice lower for RED-IBTMS and more than five times lower for RED-OTES compared to the unmodified pigment. Thus, a stronger effect on the size of the formed aggregates was observed in the case of pigments modified with octyltriethoxysilane OTES.

**Figure 4:**
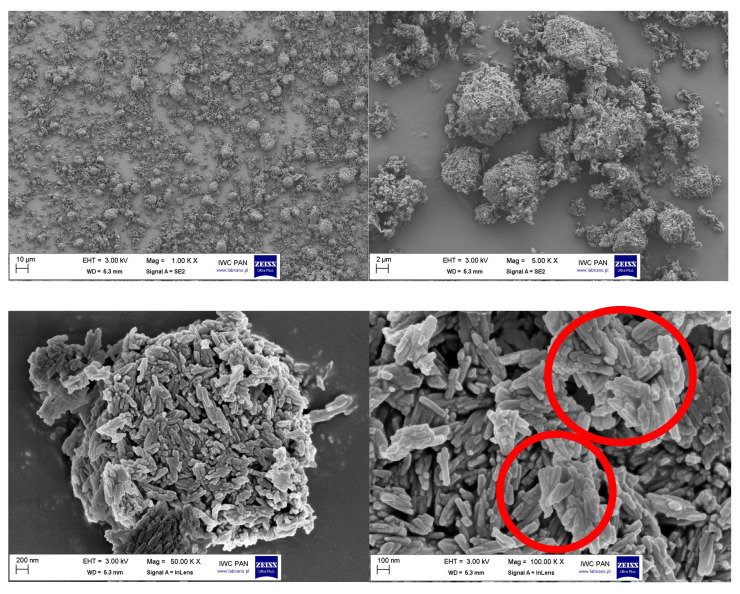
The SEM pictures of pigment RED 122 at magnifications of 1000, 5000, 50,000 and 100,000 times (SEM microscope, LEO 1530 Gemini, producent Zeiss/Leo, Oberkochen, Germany). Red circles mark the stacked together large pigment aggregates.

In addition to the DLS method, the influence of silane modification on the break-up of the pigment RED 122 aggregates was analyzed using SEM microscopy. In Figure 4, the SEM pictures of pigment RED 122 are shown. The primary particles of unmodified pigments are stacked together forming large aggregates, as marked on the SEM image.

**Figure 5:**
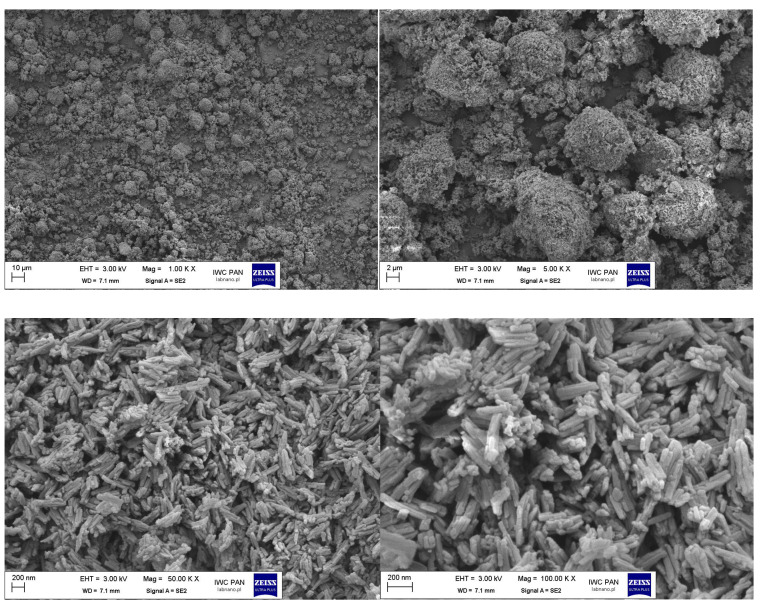
The SEM pictures of pigment RED 122 modified with 1 wt% of isobutyltrimetoxysilane IBTMS at magnifications of 1000, 5000, 50,000 and 100,000 times (SEM microscope, LEO 1530 Gemini, producent Zeiss/Leo, Oberkochen, Germany).

The pulverization method, together with silane modification using a ball mill, led to the destruction of the largest pigment aggregates, as shown for the RED 122 pigment modified with 1 wt.% of isobutyltrimetoxysilane IBTMS (Figure 5). The SEM pictures for the pigment modified with 2 wt.% of IBTMS and with 1 wt.% and 2 wt% of octyltriethylosilane OTES are compiled in Supplementary Materials’ Figures S5–S7. Here, the modification also led to the break-up of the pigment aggregates.

After modification with selected silanes, the total surface free energy of pigment RED 122 changed (Table 3). The covering of the pigment surface with both IBTMS and OTES silanes strongly influenced the polar component of the surface energy γ_p_. This confirmed that both silanes were able to interact with polar groups of pigment RED 122. Furthermore, the presence of non-polar alkyl groups caused the enhancement in the dispersive component of surface free energy γ_d_ as compared with the unmodified pigment. It is worth noting the strong decrease in pigment polarity γ_p_/γ after modification with respect to the unmodified pigment RED 122, which is beneficial from the point of view of mixing them with non-polar polymers such as polyolefins. Reduced polarity can facilitate the mixing of modified pigment with polyethylene, leading to a more homogenous dispersion of modified pigments in matrix.

**Figure 6:**
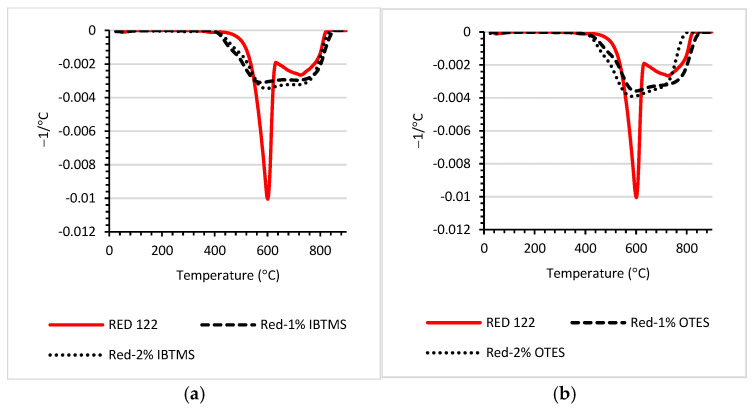
dTGA plots of silane-modified RED 122 pigment: (**a**) pigment modified with 1 and 2 wt.% of IBTMS; (**b**) pigment modified with 1 and 2 wt.% of OTES.

Thermal stability of the modified pigments was analyzed using the TGA method (Figure 6). The temperatures at 5, 10, 20 and 50% of weight loss for the studied pigments are compiled in Table 4. The dTGA plots for unmodified RED 122 confirmed the high thermal stability of the pigment. The degradation and weight loss for the RED 122 pigment occurred in the temperature range of 450–820 °C (Figure 6). Two-peaks in the DTGA plots were identified, indicating a two-step mechanism of weight loss, and the maximum of the peaks was at 601 °C and 733 °C. For the silane-modified RED 122 pigment, one broad peak was observed on dTGA plots. Both OTES and IBTMS affected the mechanism of pigment weight loss. Further, for the silane-modified pigments, weight loss started faster, and 5% and 10% of weight loss were determined at a lower temperature. Lower thermal stability is not disadvantageous when the silane-modified pigments are used as a coloring substance for polyethylene; the temperature of 1% weight loss for every silane-modified pigment was determined much above the processing temperatures of polyethylene.

The influence of the modification on the color profile of the pigment is very important. The CIELab* parameters for modified pigments are compiled in Table 5. The modification with isobutyltrimetoxysilane IBTMS shifted the color coordinate a toward a stronger red color and coordinate b toward a stronger blue color. Opposite changes in the a and b coordinates were observed after modification with octyltrietoxysilane OTES, the deeper shades of green and yellow were determined for OTES-modified pigments as compared to pigment RED 122.

**Figure 7:**
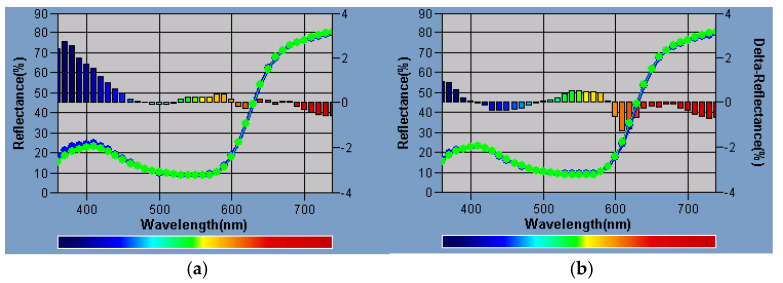
The reflectance plots for RED-1% IBTMS (**a**) and RED-1% OTES pigment (**b**). Blue curve – the reference Pigment RED 122, Green plot modified pigment, respectively RED-1% IBTMS (**a**), RED-1% OTES (**b**).

Figure 7 shows the differences in the color profile between pigment RED 122 and the pigment modified using 1 wt.% of isobutyltrimetoxysilane IBTMS or 1 wt.% of octyltriethoxysilane OTES. The reflectance (%) plots for RED-2% IBTMS and RED-2% OTES are compiled in Supplementary Materials’ Figures S8 and S9. The modification with 1 wt.% of both silanes slightly influenced the color profile of pigment RED 122, which was also confirmed by the low values of the ΔE parameter (Table 5).

### 3.2. Dynamic Viscoelastic Shear Properties of Colored Masterbatches

The rheological behavior of the coloring concentrates and the effect of 2 wt.% of the concentrate on the viscoelastic properties of the masterbatch/polyethylene mixtures were investigated at 170 °C. The Ares G2 oscillation rotational rheometer was used during the studies. The measurements were carried out at a variable strain amplitude as well as at a variable frequency. Additionally, oscillation measurements were used to analyze the behavior of the material during melting and to determine the melting temperature T_m_.

#### 3.2.1. Viscoelastic Properties of Colored Masterbatches at 170 °C as a Function of Oscillation Strain

**Figure 8:**
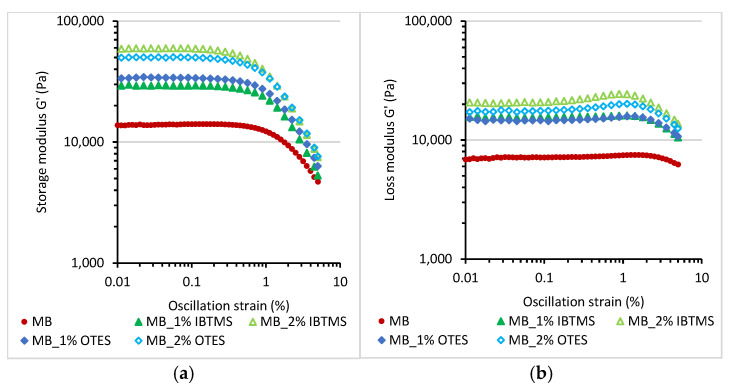
The storage shear modulus G′ (**a**) and loss shear modulus (**b**) measured at 170 °C as a function of oscillation strain for the masterbatches containing pigment RED 122 and IBTMS and OTES silane-modified pigments; the angular frequency applied was 10 rad·s^−1^.

Figure 8 shows the values of the storage shear modulus G′ and loss shear modulus G″ for melted colored masterbatches determined as a function of oscillation strain up to 5% using a constant value of angular frequency of 10 rad·s^−1^ and a temperature of 170 °C.

The viscoelastic behavior of the concentrated mixtures of pigments in the polymeric carrier is different as compared with that of pure polyethylene PE or 2 wt.% of concentrate in PE (Supplementary Materials, Figure S10). Both PE and the melted mixtures of PE and 2 wt.% of prepared concentrate showed linear viscoelastic behavior up to 5% of oscillation strain. No significant changes in the values of storage shear modulus G′ and loss shear modulus G″ were observed in studied range of oscillation strain. In contrast, for the melted colored masterbatches, a shorter linear viscoelastic region LVR and strong influence of the applied oscillation strain on the viscoelastic parameters were observed (Figure 8). For all masterbatches, the strong reduction in value of the storage shear modulus G′ for an oscillation strain stronger than 1% was observed. Moreover, the modification of pigment RED 122 by both silanes led to increase in the storage shear modulus G′ of the melted masterbatches. The use of a higher amount of modifying silane substance enhanced this effect. The increase in G′ was influenced by a reduction in the size of pigment aggregates due to pulverization grinding, which, in turn, improved the dispersion of the pigment in the polymer. Additionally, an internal structure was formed through particle–particle interactions.

Different viscoelastic behavior of masterbatches with respect to the polyethylene carrier, and much higher values of storage shear modulus G′_LVR_, loss shear modulus G″_LVR_ and complex modulus G*_LVR_ determined for linear viscoelastic region resulted from a high concentration of the added pigment acting as a reinforcing filler (Table 6). Furthermore, silane modification led to an enhancement in the storage shear modulus G′LVR of the modified pigments masterbatches. Also, the viscoelastic parameters of the polyethylene samples containing 2 wt.% of MB_IBTMS and MB_OTES masterbatches were higher as compared with those of polyethylene containing the unmodified pigment.

The dynamic properties of filled polymeric systems are affected by various factors. Among them, the following are very important: (a) the size of the formed aggregates, (b) the possibility of creation of an internal structure by solid particles via particle–particle interactions, and (c) the interaction between the filler and the polymer chains [53]. These factors can enhance the values of the storage shear modulus G′ and influence the loss shear modulus G″. A better dispersion of filler particles, and smaller aggregates formed in the polymeric matrix led to an improvement in the viscoelastic properties and a reinforcing effect, as observed for silane-modified silica in rubber [54].

Pigment RED 122 can act as a reinforcing solid additive if a homogenous, at a nanometric scale, dispersion of the pigment particles is achieved. Furthermore, this pigment is able to form hydrogen bonds via polar groups present in the structure, promoting the formation of an internal particle–particle structure [17]. The modification of the pigment particles changed the surface free energy of the pigment, facilitated the mixing with the polymer, and reduced the diameter of the formed aggregates, improving the homogeneity of its dispersion throughout the polyethylene carrier, resulting in changed viscoelastic properties as compared with those of the masterbatch containing the unmodified pigment. The improvement in pigment dispersion after modification is responsible for the observed increase in G′, G″, G*_LVR_ moduli (Table 6) with respect to those of samples containing the unmodified pigment.

The other factor that can strongly affect the viscoelastic properties of masterbatches containing a high concentration of modified pigments is the tendency to create a particle structure in the carrier. Particle–particle interactions are one of the factors enhancing Payne’s effect and leading to an increase in the storage shear modulus for filled polymeric systems [55]. The internal solid particle structure formed due to the applied deformation being higher is usually broken, causing a reduction in the G′ and G* values, as observed for the reinforcing fillers such as silica or carbon black in elastomers [53,55,56]. The break-up of the structure usually occurs up to 5% of oscillation strain [53].

In Table 7, the values of storage shear modulus G′ and complex modulus G* measured for the colored masterbatches at the 0.01% and 5% of oscillation strain and the calculated parameters ΔG′ and ΔG* are compiled. Higher values of ΔG′ and ΔG* indicate a stronger Payne’s effect and a stronger tendency to create particle–particle structure. The modification with both silanes promoted Payne’s effect, and a stronger reduction in the values of G′ and G* was observed due to the break-up of the internal particle–particle structure.

**Figure 9:**
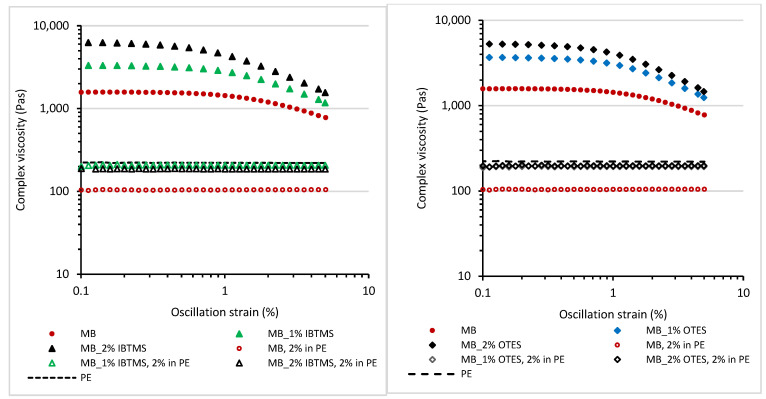
Complex viscosity η* measured at 170 °C as a function of oscillation strain for the masterbatches containing pigment RED 122 and silane-modified pigments; the angular frequency applied was 10 rad·s^−1^.

The very important rheological parameters influencing the processing of melted polymers are the complex viscosity η* and the values of the loss factor tan δ (Figure 9, Table 8).

As expected, the incorporation of a high amount of pigment increased the complex viscosity η* (Table 8). Further, the modification of the pigment and the improvement in pigment dispersion had an additional impact on the viscosity η*of melted masterbatches. Smaller aggregates and stronger modified pigment particle interactions led to an increase in the complex viscosity η* as compared with the masterbatch based on the unmodified pigment. For all masterbatches, a shear thinning behavior was observed (Figure 9). The stronger applied oscillation strain (higher shear rate) caused the reduction in complex viscosity. The effect was stronger for the masterbatches containing silane-modified pigments.

From an industrial point of view, the addition of colored concentrate should not significantly influence the viscosity η* of molten polymer during the processing and production of the colored product. The observed complex viscosity η* of PE mixture with 2 wt.% of masterbatch containing the unmodified pigment is lower than that of the unmodified polyethylene (Figure 9). In contrast, the complex viscosity η* of the polyethylene containing 2 wt.% of masterbatches based on silane-modified pigments is almost similar to the η* of melted polyethylene used as a carrier. This is advantageous. The optimized level of viscosity allows us to avoid processing problems resulting in a non-uniform color of the product.

**Figure 10:**
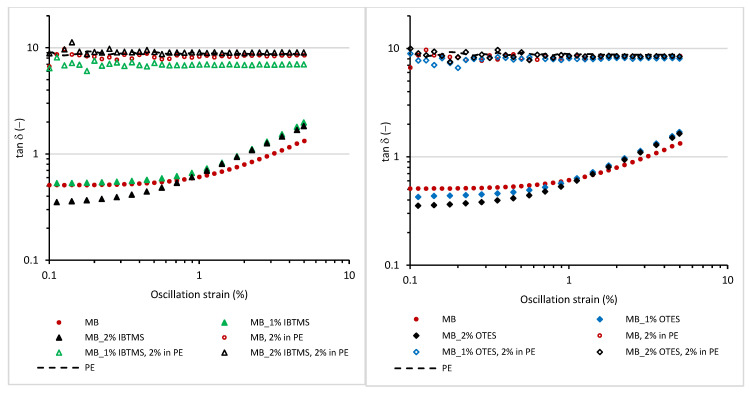
Loss factor tan δ measured at 170 °C as a function of oscillation strain for the masterbatches containing pigment RED 122 and silane-modified pigments; the angular frequency applied was 10 rad·s^−1^.

The loss factor tan δ (Figure 10) of masterbatches containing pure pigment RED 122 and silane-modified pigment is much lower than the tan δ determined for polyethylenes containing 2 wt.% of masterbatches. Moreover, in the oscillation strain range studied (shear deformation up to 5%), no significant changes in the values of tan δ were observed. In contrast, the tan δ of masterbatches increased as the applied oscillation strain was higher. It should be taken under consideration that masterbatches contain a high concentration of pigment (30 wt.%). The presence of solid pigment particle influences the dissipation of energy. As we previously observed, the application of higher deformation led to a reduction in storage shear modulus G′ resulting from the destruction of pigment particle–particle structure. Hence, a decrease in tan δ values is observed as the applied deformation is higher.

#### 3.2.2. Viscoelastic Properties of Colored Masterbatches at 170 °C as a Function of Angular Frequency and Temperature

**Figure 11:**
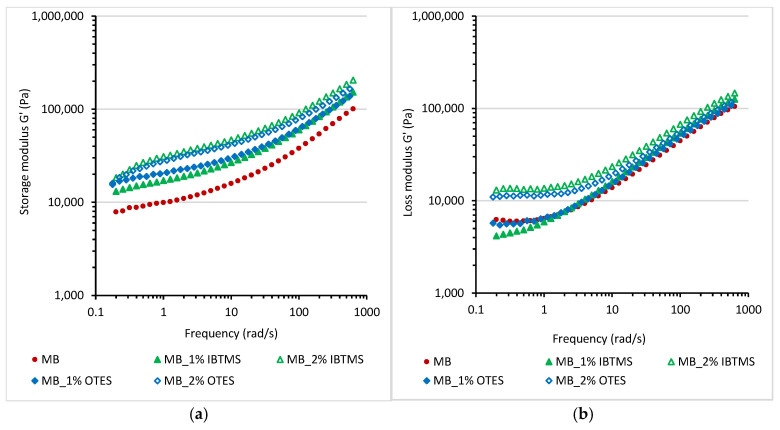
Storage shear modulus G′ (**a**) and loss shear modulus G″ (**b**) measured at 170 °C as a function of angular frequency for the masterbatches containing pigment RED 122 and silane-modified pigments; the oscillation strain applied was 0.5%, and the temperature was 170 °C.

The viscoelastic properties of the obtained masterbatches were investigated as a function of angular frequency (Figure 11). For all masterbatches, the values of storage modulus G′ were higher than the values of loss modulus G″ in the frequency range studied. Further, the values of storage shear modulus G′ and loss shear modulus G″ increased after the use of silane-modified pigments in the masterbatch formulation. This is attributed to the reduction in size of the pigment agglomerates and a more homogenous dispersion in the carrier. The values of storage modulus G′ at low values of the applied frequency were higher for the masterbatches containing modified pigments. Higher values of storage modulus G′ at a low frequency may lead to stronger post-extrusion swelling during processing [45].

In contrast to the masterbatches, for melted mixtures of 2 wt.% of masterbatches and polyethylene, the values of loss shear modulus G″ were higher than the values of storage shear modulus (Figure S10, Supplementary Materials). Furthermore, the addition of masterbatches containing silane-modified pigments increased the values of storage shear modulus G′. A stronger decrease in G′ and G″ as the applied frequency was lower was observed for the melted mixtures containing modified pigments, indicating facilitated relaxation.

To estimate the impact of 2 wt.% of masterbatches containing modified pigments on the relaxation of the melted polyethylene, the continuous relaxation model [57,58,59] described by Equations (4) and (5) was applied and the spectrum H(lnτ) was obtained by fitting the oscillation data to Equations (4) and (5) [58,59].

(4) $$G^{\prime }\left(\omega \right)=\int_{-\omega }^{+\omega }H\left(ln\tau \right)\frac{\omega^{2}\tau^{2}}{1+\omega^{2}\tau^{2}}dln\tau$$

(5) $$G^{\prime \prime }\left(\omega \right)=\int_{-\omega }^{+\omega }H\left(ln\tau \right)\frac{\omega \tau }{1+\omega^{2}\tau^{2}}dln\tau$$

where G′—storage shear modulus; G″—loss shear modulus; and [H_i_, τ_i_] are the pairs of fitted relaxation parameters.

**Figure 12:**
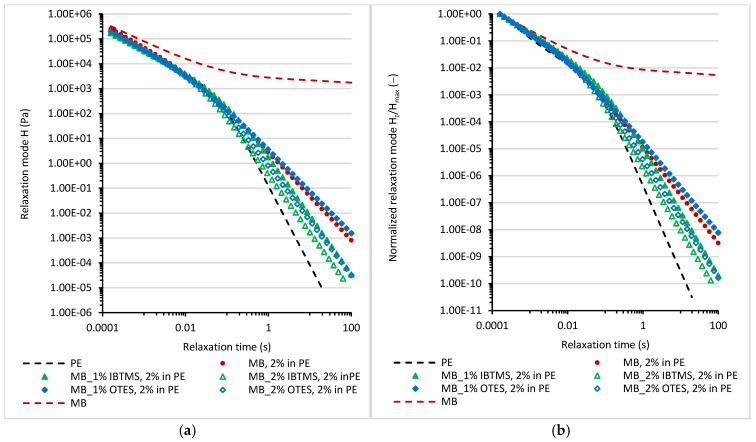
Continuous relaxation spectra for polyethylene samples containing 2 wt.% of various masterbatches melted at 170 °C (**a**). Normalized relaxation spectra H_i_/H_max_ = f(τ_i_) for melted samples containing 2 wt.% of masterbatches (**b**).

In Figure 12, the parameters [H_i_,τ_i_] calculated using the continuous relaxation model and the normalized relaxation plot H_i_/H_max_ = f(τ_i_) are shown. Additionally, for comparison, data calculated for the unmodified polyethylene and the masterbatch containing pigment RED 122 were added to the plots. The relaxation of the masterbatch due to the presence of a high concentration of pigment particles is slower. The incorporation of 2 wt.% of the masterbatch into polyethylene influenced relaxation. For all studied samples, longer relaxation times were calculated as compared with unmodified melted polyethylene. The modification of pigment particles with isobutyltrimetoxysilane IBTMS facilitated relaxation. Shorter relaxation times τ are observed for melted samples containing 2 wt.% MB-IBTMS in polyethylene as compared with those containing RED 122 pigment. In case of modification using octyltrietoxysilane OTES, this effect is observed only for the MB_2% OTES, 2% in PE sample. The modification of pigment RED 122 with a higher concentration of octyltriethoxysilane led to the facilitated relaxation of MB_2% OTES, 2% in PE as compared with MB, 2% in PE. No significant changes in relaxation mode between samples MB, 2% in PE and MB-1% OTES, 2% in PE were observed.

Based on the temperature sweep oscillation tests for a temperature range of 110–200 °C, the cross-points of the storage shear modulus G′ and loss shear modulus G″ indicating melting of the material were determined. The results of these analysis are compiled in Table 9.

No significant differences in melting temperatures between samples were observed; the cross-points temperatures determined were in range of 120–123 °C, with the highest being observed for masterbatch containing pigment RED 122 (123.20 °C) and the lowest for the masterbatch containing pigment modified with 1 wt.% of octadecyltriethoxysilane OTES (120.92 °C). For all masterbatches, the values of storage G′ and loss modulus G″ at the cross-point were higher as compared with the polyethylene samples containing 2 wt.% of color concentrates. This was a result of the higher concentration of solid particles (pigment) in the melted polymer. The values of G′ and G″ moduli at the cross-point for polyethylene samples containing 2 wt.% of OTES and IBTMS color concentrates decreased. This is advantageous as lower values of the moduli at the melting point facilitate processing. The plots of storage shear modulus G′ and loss shear modulus G″ as a function of temperature for the studied samples are compiled in Supplementary Materials’ Figures S11 and S12.

**Figure 13:**
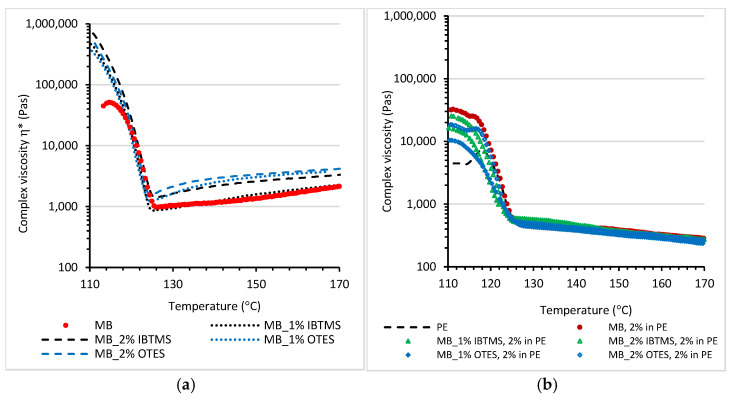
Complex viscosity η* measured as a function of temperature for the masterbatches containing pigment RED 122 and silane-modified pigments (**a**), and for the polyethylene containing 2 wt.% of added masterbatches (**b**); the applied angular frequency was 10 rad·s^−1^, the applied oscillation strain was 0.02%, and the heating rate was 5 °C·min^−1^.

Additionally, in Figure 13, the values of complex viscosity η* for masterbatches containing pigment RED 122 and silane-modified pigments (a), and for the polyethylene containing 2 wt.% of masterbatches (b) are shown.

The complex viscosity η* of masterbatches containing silane-modified pigments was significantly higher in the whole temperature range studied, which was a result of the improvement in the dispersion and deagglomeration of pigment particles, as described in the previous section. Important from an industrial point of view and further application of modified masterbatches during the processing of colored products is that the masterbatches containing modified pigments after melting at temperatures higher than 130 °C did not cause a strong enhancement in complex viscosity η* after mixing with polyethylene.

### 3.3. Rotational Test of Melted Colored Masterbatches

**Figure 14:**
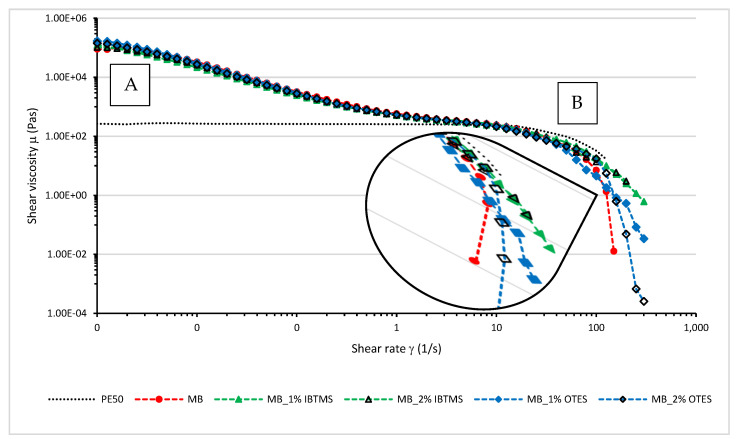
Shear viscosity μ of masterbatches containing RED 122 pigment and pigments modified by silanes. The rotational tests were carried out at 170 °C as a function of the applied shear rate in range of 0.001–300 s^−1^. A—the Newtonian plateau for the pure polyethylene; B—the zone of shear thinning behavior of the pure polyethylene.

Rotational rheological measurements were carried out and steady flow sweep tests were performed to determine shear viscosity µ as a function of the shear rate $\dot{\gamma }$ In Figure 14, the values of shear viscosity µ measured at 170 °C as a function of shear rate in the range of 0.001–300 s^−1^ for melted colored masterbatches are depicted. For comparison, the values of shear viscosity determined for the polymeric polyethylene carrier used to prepare the masterbatches were added to the plot. Two ranges of shear rate are marked in the plots, zone A (the shear rate from 0.001 s^−1^ up to 5 s^−1^) and zone B (the shear rate from 50 s^−1^ up to 300 s^−1^). In zone A, for the unmodified polyethylene used as a carrier for masterbatches, a Newtonian plateau was observed with a constant value of shear viscosity. When the applied shear rate increased and reached values higher than 10 s^−1^, the shear viscosity of melted polyethylene decreased. Shear thinning behavior and a strong reduction in shear viscosity were observed for zone B and values of the shear rate higher than 30 s^−1^. The level of shear rate from 50 to 300 s^−1^ is typical for processing methods such as compression molding and extrusion. In contrast to the polyethylene carrier, for all color concentrates, shear thinning behavior is observed at low values of the shear rate (zone A). The reduction in viscosity that occurred in zone A was due to the break-up of the pigment particle structure formed via particle–particle interactions. This confirmed the observation from the oscillation amplitude sweep tests. In zone B, the profiles of shear viscosity did not significantly change from polyethylene; however, a similar reduction in viscosity occurred for different levels of the shear rate. Especially of note, for the masterbatches containing modified pigments as compared with the masterbatch based on the pigment RED 122, higher values of the shear rate were needed to achieve a similar lever of viscosity.

**Figure 15:**
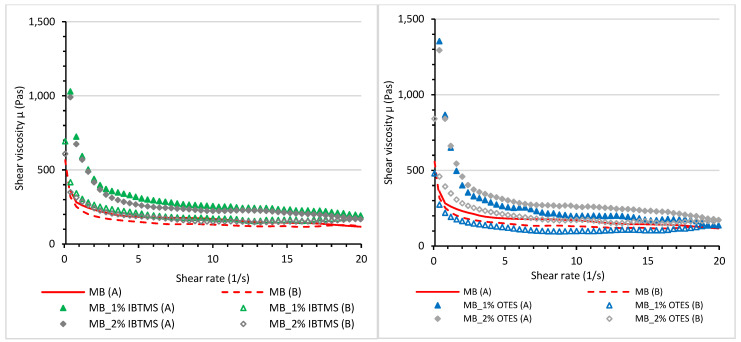
Shear viscosity μ of masterbatches containing RED 122 pigment and pigments modified by IBTMS and OTES silanes measured at 170 °C as a function of the shear rate. Index A in the graph means that the viscosity was measured using an increasing shear rate; index B means that the viscosity was measured using a decreasing shear rate.

To analyze the differences in pigment particle structurization in melted color concentrates, viscosity hysteresis loops were measured at 170 °C. In Figure 15, the values of shear viscosity measured using two steps are shown; in the first step (A), the applied shear rate increased in the range of 0.01–20 s^−1^ for 60 s; in the second step (B), the applied shear rate was in the range of 20–0.01 s^−1^. Figure S13 in the Supplementary Materials shows the difference in the viscosity loops between the polyethylene carrier and the masterbatch containing pigment RED 122. The calculated area under the viscosity plots and the determined area of the viscosity loops are compiled in Table 10.

The modification of pigment surface using isobutyltrimethoxysilane IBTMS or octyltriethoxysilane OTES increased the structurization of the masterbatches, resulting in a larger area of the viscosity loops, as shown in Figure 15. The internal structure formed via modified pigment particle–particle interactions was easily broken as the applied shear rate increased. The areas of the viscosity loops were larger for the masterbatches containing the pigment modified with octyltrietoxysilane OTES (Table 10).

The shear viscosity µ as a function of the shear rate $\dot{\gamma }$ was also determined for the melted polyethylene containing 2 wt.% of obtained masterbatches (Supplementary Materials Figure S14). The determined data of shear viscosity μ_i_ were fitted to two models: 1) the Carreau–Yasuda viscosity model, according to Equation (6); and the Carreau model, according to Equation (7) [60]:

(6) $$\frac{\mu_{i}-\mu_{\infty }}{\mu_{0}-\mu_{\infty }}=[1+(k{\dot{\gamma }}_{i}{)}^{a}{]}^{\frac{n-1}{a}}$$

where μ_0_ is the zero shear viscosity, μ_∞_ is the infinite viscosity, k is the consistency (characteristic time), and n is the power law index and a parameter describing the transition between Newtonian plateau and the power law region.

(7) μi−μ∞μ0−μ∞=1(1+(kγ˙i)2)n2

where μ_0_ is the zero shear viscosity, μ_∞_ is the infinite viscosity, k is the consistency (characteristic time), and n is the power law index.

The calculated parameters are compiled in Table 11. For all polyethylene mixtures containing silane-modified pigments, higher levels of correlation R^2^ (0.997–0.999) were achieved by fitting the viscosity data to the Carreau–Yasuda model. For the polyethylene mixture containing the RED 122 pigment, the level of correlation with the Carreau–Yasuda model was R^2^ = 0.994; thus, the Carreau model was applied (R^2^ = 0.999). Modification of pigment RED 122 with selected silanes and subsequent use of such pigments as a component of the masterbatch introduced in the amount of 2 wt.% into polyethylene resulted in an increase in zero viscosity μ_0_. It was a result of a more homogenous dispersion of modified pigments with smaller pigment aggregates, as further confirmed by SEM analysis. The higher values of the n parameter (power law index), indicating a stronger decrease in shear viscosity as an applied shear rate, were higher for the melted polyethylene containing 2 wt.% of the unmodified pigment RED 122 masterbatch (MB). Moreover, for sample MB 2 wt.% in PE, the value of the calculated the infinite viscosity μ_∞_ was lower. The modification with IBTMS and OTES silanes influenced consistency k. Characteristic times of melted at 170 °C colored polyethylene increased after incorporation of 2 wt.% of color concentrates containing silane-modified pigments indicating that relative mobility or ability to flow was lower.

### 3.4. Influence of Colored Concentrates on Thermal Properties and Crystallization of Polyethylene

**Figure 16:**
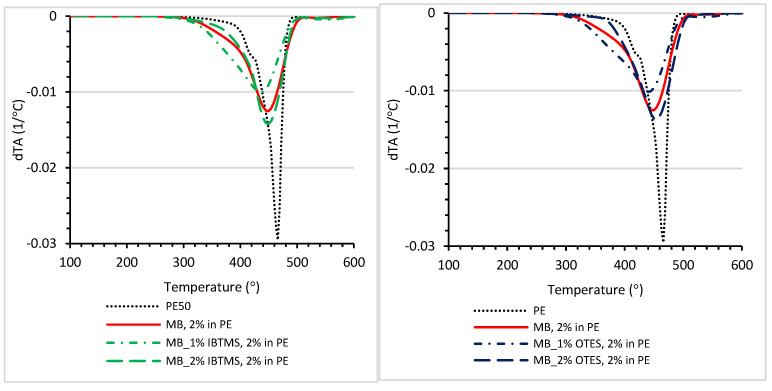
dTGA plots of polyethylene containing 2 wt.% of pigment RED 122 color concentrate (MB) and IBTMS silane-modified RED 122 pigment or 2 wt.% of color concentrate containing pigment modified with OTES.

A TGA analysis of polyethylene containing 2 wt.% of colored substances was carried out. In Figure S15 in the Supplementary Materials, plots of weight loss (%) measured as a function of temperature are compiled. The dTA plots are shown in Figure 16. 

The temperatures of 5, 10, 20% of weight loss and the temperature at the maximum of the dTA peak are compiled in Table 12.

The incorporation of color concentrates shifted the dTA peak towards lower values of temperature (Figure 16); the lower values of temperature T_max_ were determined at the maximum of the peak (Table 12) as compared with unmodified polyethylene PE. Only for the polyethylene mixture containing 2 wt.% of color concentrate MB_2%-OTES was the T_max_ higher as compared with that of MB, 2 wt.% in PE. Here, the modification of pigment RED 122 using 2 wt.% of octadecylsilane led to an enhancement in the thermal properties of colored polyethylene mixtures. From an industrial point of view, it is important that 1 and 5% of weight loss (T_1%_, T_5%_) occurred at a relatively high temperature, higher than 300 °C. Pigment RED 122 is primarily used in polyolefins. However, it can be used in other polymers whose processing temperature reaches up to 270 °C. Therefore, the thermal stability of the pigment is required to be higher than the mentioned temperature, and no weight loss should be observed.

To estimate the influence of the additives on crystallization temperature T_c_, melting temperature T_m_ and degree of crystallinity χ_c_, DSC measurements were performed. The results of DSC studies are compiled in Table 13. The examples of DSC plots are shown in Supplementary Materials’ Figures S16–S18.

The presence of pigment RED 122 particles or IBTMS and OTES silane-modified pigment influenced the crystallization of polyethylene (Table 13). The calculated degree of crystallinity increased as compared with that of unmodified polyethylene. The presence of pigment particles was the main factor influencing the crystallization of polyethylene. Furthermore, in most cases, the modification of pigment particles reduced the nucleating effect of pigment particles, leading to a lower degree of crystallinity χ_c_ as compared to that of samples containing pure RED 122 pigment. The incorporation of pigment RED 122 or modified pigment shifted the crystallization temperatures T_c_ towards higher values of temperature. Here, the effect was significant, and the crystallization temperatures T_c_ shifted from ~107 °C (polyethylene) up to 113–114 °C (polyethylene containing coloring substances). The influence of pigments on the melting temperature T_m_ was not significant. The melting temperatures were in range of 122–124 °C. A similar range of melting temperatures was determined using oscillation temperature sweep tests.

### 3.5. SEM Analysis of Color Concentrates

**Figure 17:**
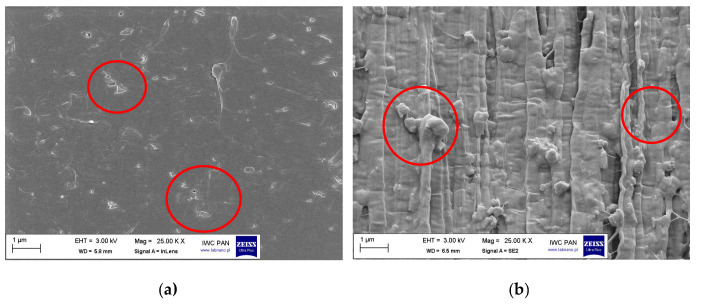
SEM pictures of pellets of masterbatch MB containing RED 122 pigment (concentration pf pigment 30 wt.%) at magnifications of 25,000 times. The pictures were taken as a cross-section of the granulate along the *X* axis (**a**) and as a cross-section along the *Y* axis of the granulate (**b**). Red circles mark the pigment aggregates and voids in material structure.

**Figure 18:**
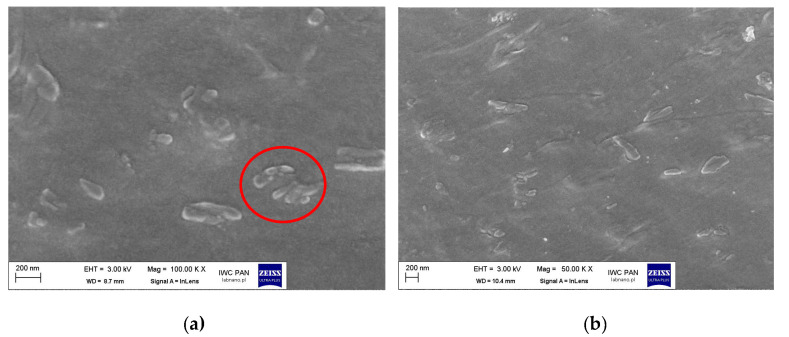
SEM pictures of pellets of masterbatch MB_IBTMS 1% (**a**) and MB_IBTMS 2% (**b**). The pictures were taken as a cross-section of the granulate along its *X* axis. Red circle marks the pigment aggregates.

**Figure 19:**
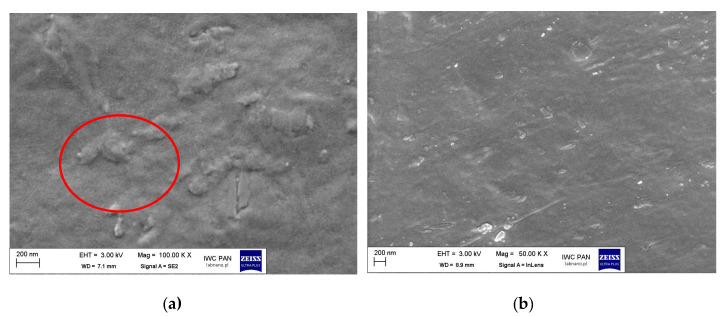
SEM pictures of pellets of masterbatch MB_OTES 1% (**a**) and MB_OTES 2% (**b**). The pictures were taken as a cross-section of the granulate along its *X* axis. Red circle marks the pigment aggregates.

The dispersion of pigment RED 122 and silane-modified pigments in masterbatches was analyzed using Scanning Electron Microscope SEM pictures (Figure 17, Figure 18 and Figure 19).

In Figure 17, images of masterbatch MB pellets containing pigment RED 122 are shown. The grains of the agglomerated pigment with a size of more than 1 μm are present in the polymeric carrier. The pigment particles are not dispersed homogenously throughout the matrix. Moreover, the SEM pictures taken as a cross-section along the *Y* axis of the pellet showed the presence of voids in the structure. An uneven distribution of pigment grains along the *Y* axis was observed.

After pigment modification with 1 wt.% of isobutyltrimethoxysilane IBTMS, the size of the aggregates formed in polyethylene pigment was lower, of less than 500 nm. Nevertheless, the pigment particles stacked together were observed as shown in Figure 18a. The reduction in aggregate size was stronger when the amount of IBTMS silane used to modify RED 122 pigment was 2 wt.%., as shown in Figure 18b.

Larger aggregates were observed for the pigment modified with 1 wt.% of OTES as compared with the pigment modified with IBTMS. The pigment aggregates were tightly surrounded by polymer material, which may indicate better adhesion of the pigment modified with OTES to the carrier matrix, as shown in Figure 19a. In this case, the use of a larger amount of OTES modifier also resulted in increased homogeneity of the pigment grain distribution in the carrier.

The analysis of SEM images confirmed that modification with selected silanes led to an improvement in pigment dispersion in the masterbatches. Furthermore, after the incorporation of modified masterbatches into polyethylene during processing, more homogenous pigment particle dispersion was also observed.

## 4. Conclusions

After performing DLS measurements, it was proven that surface modification of the PR 122 IBTMS and OTES pigment reduces the range of aggregates formed by the pigment. This trend was also confirmed in the SEM images, where the disintegration of the largest pigment aggregates was noted. Additionally, the modification caused a decrease in the polarity of the pigment, which is an advantage when coloring non-polar polymers such as polyethylene. On the other hand, it contributed to its slightly lower thermal stability. Small changes in the L*a*b* parameters were also observed, and the greatest differences are seen when 2% silanes are introduced into the crude pigment.

The introduction of the silane-modified pigment into the masterbatch increases the values of the viscoelastic parameters and promotes the Payne effect. The larger hysteresis loops of the new masterbatches with silanes indicate greater structuring of coloring concentrates.

The introduction of the masterbatch into polyethylene causes an increase in the temperature of crystallization T_c_ and degree of crystallinity compared to those of pure polyethylene.

## Figures and Tables

**Table 1:** The diameter of pigment RED-122 aggregates formed in the polar water medium.

Sample	Range of Aggregate Size (nm) *	Size of the Main Fraction (nm) *	Percentage as Number of Main Fraction (%) *
Pigment RED 122	122–1106	220.2	15.9
RED-1% IBTMS	164–955	342.0	17.8
RED-2% IBTMS	255–825	458.7	26.3
RED-1% OTES	51–396	91.3	22.7
RED-2% OTES	79–459	141.8	22.4

* Measurements were taken for the dispersion of pigments in the polar water medium.

**Table 2:** The diameter of pigment RED-122 aggregates formed in the non-polar paraffin oil medium.

Sample	Range of Aggregate Size (nm) *	Size of the Main Fraction (nm) *	Percentage by Number of Main Fraction (%) *
Pigment RED 122	3580–7456	5560	41.6
RED-1% IBTMS	1106–4145	1990	26.5
RED-2% IBTMS	1281–4801	2669	24.6
RED-1% OTES	712–2305	1281	32.7
RED-2% OTES	459–1281	712	31.4

* Measurements were taken for the dispersion of pigments in the non-polar paraffin oil medium.

**Table 3:** Calculated values of surface free energy, *γ*, dispersive, *γ*_d_, and polar, *γ*_p_ components of surface free energy and the polarity index, *γ*_p_/*γ*.

Sample	*γ*_p_ mN·m^−1^	*γ*_d_ mN·m^−1^	*γ* mN·m^−1^	*γ*_p_/*γ*
Pigment RED 122	1.0	11.9	12.8	0.078
RED-1% IBTMS	0.1	21.1	21.2	0.005
RED-2% IBTMS	0.3	15.0	15.3	0.020
RED-1% OTES	0.2	15.6	15.9	0.013
RED-2% OTES	0.1	11.7	11.8	0.008

**Table 4:** The temperatures at 5, 10, 20 and 50% of weight loss, T_5%_, T_10%_, T_20%_, and T_50%_.

	T_5%_ (°C)	T_10%_ (°C)	T_20%_ (°C)	T_50%_ (°C)
Pigment RED 122	532	559	583	631
RED-1% IBTMS	468	506	548	646
RED-2% IBTMS	478	510	557	646
RED-1% OTES	482	515	562	646
RED-2% OTES	464	496	538	618

**Table 5:** Average values of color profile of modified pigment.

	L	a	b	ΔE
Pigment RED 122	44.01	36.25	−5.99	---
RED-1% IBTMS	44.23 (0.22)	36.38 (0.13)	−6.61 (−0.62)	0.67
RED-2% IBTMS	42.56 (−1.46)	38.14 (1.88)	−6.77 (−0.78)	2.51
RED-1% OTES	44.16 (0.15)	34.76 (−1.49)	−5.15 (0.83)	1.71
RED-2% OTES	43.97 (-0.04)	34.21 (−2.04)	−5.12 (0.86)	2.21

**Table 6:** Storage shear modulus G′_LVR_, loss shear modulus G″_LVR_, and complex modulus G*_LVR_ measured for linear viscoelastic region (LVR) at 170 °C.

	G′_LVR_ (kPa)	G″_LVR_ (kPa)	G*_LVR_ (kPa)
Polyethylene	0.25 ± 0.01	2.21 ± 0.02	2.22 ± 0.02
MB	13.83 ± 0.13	7.05 ± 0.13	15.52 ± 0.13
MB, 2% in PE	0.14 ± 0.04	1.00 ± 0.06	1.02 ± 0.05
MB_1% IBTMS	29.24 ± 0.17	15.54 ± 0.16	33.11 ± 0.17
MB_1% IBTMS, 2% in PE	0.293 ± 0.09	2.05 ± 0.01	2.07 ± 0.01
MB_2% IBTMS	58.74 ± 0.82	20.67 ± 0.30	62.27 ± 0.78
MB_2% IBTMS, 2% in PE	0.20 ± 0.09	1.86 ± 0.02	1.87 ± 0.02
MB_1% OTES	33.79 ± 0.354	14.52 ± 0.38	36.78 ± 0.38
MB_1% OTES, 2% in PE	0.243 ± 0.02	1.92 ± 0.0	1.93 ± 0.01
MB_2% OTES	49.67 ± 0.44	17.46 ± 0.32	52.65 ± 0.43
MB_2% OTES, 2% in PE	0.24 ± 0.01	1.96 ± 0.02	1.97 ± 0.02

Applied angular frequency: 10 rad·s^−1^; temperature: 170 °C.

**Table 7:** Complex shear modulus G*, storage shear modulus G′ measured for 0.01 and 5% of oscillation strain.

	G*_0.01_ kPa	G*_5%_ kPa	ΔG* kPa	G′_0.01_ kPa	G′_5%_ kPa	ΔG′ kPa
MB	15.629	7.784	7.845	14.186	4.687	9.499
MB 1% IBTMS	32.290	11.763	20.527	28.734	5.298	23.436
MB 2% IBTMS	59.765	15.643	44.122	56.575	7.492	49.083
MB 1% OTES	36.274	12.458	23.816	34.215	6.338	27.877
MB 2% OTES	52.753	14.647	38.106	49.628	7.631	41.997

Values of ΔG* and ΔG′ were calculated according to ΔG* = G*_ 0.01_ − G*5% and ΔG′ = G′0.01 − G′_ 5%_. During test, an angular frequency of 10 rad⋅s^−1^ and a temperature of 170 °C were applied.

**Table 8:** Loss factor tan δ_LVR_ and complex viscosity η*_LVR_ measured for linear viscoelastic region (LVR) at 170 °C. Angular frequency applied: 10 rad·s^−1^; temperature: 170 °C.

	Tan δ_LVR_ (-)	η*_LVR_ (Pas)
Polyethylene	8.91 ± 0.44	222.2 ± 2.1
MB	0.510 ± 0.010	1552 ± 13
MB, 2% in PE	8.57 ± 2.09	101.32 ± 5.40
MB_1% IBTMS	0.531 ± 0.006	3311.24 ± 17.20
MB_1% IBTMS, 2% in PE	6.995 ± 0.219	206.63 ± 1.08
MB_2% IBTMS	0.352 ± 0.007	6226.53 ± 77.51
MB_2% IBTMS, 2% in PE	9.291 ± 0.528	186.96 ± 1.68
MB_1% OTES	0.430 ± 0.011	3678.17 ± 37.74
MB_1% OTES, 2% in PE	7.978 ± 0.460	193.33 ± 1.40
MB_2% OTES	0.352 ± 0.006	5264.70 ± 43.14
MB_2% OTES, 2% in PE	8.338 ± 0.489	196.98 ± 2.04

**Table 9:** Values of storage shear modulus G′, loss shear modulus G″ and temperature T at cross-points.

	G′ = G″ * (kPa)	T * (°C)
Polyethylene	21.64 ± 2.83	121.70 ± 0.10
MB	37.48 ± 3.58	123.20 ± 0.40
MB, 2% in PE	21.62 ± 7.23	122.19 ± 0.24
MB_1% IBTMS	33.05 ± 0.37	121.89 ± 0.04
MB 1% IBTMS, 2% in PE	8.40 ± 2.53	121.75 ± 0.11
MB_2% IBTMS	43.90 ± 0.74	122.42 ± 0.03
MB 2% IBTMS, 2% in PE	13.20 ± 4.69	121.49 ± 0.68
MB_1% OTES	48.50 ± 0.45	120.92 ± 0.03
MB 1% OTES, 2% in PE	8.99 ± 0.50	121.68 ± 0.18
MB_2% OTES	45.62 ± 0.30	121.80 ± 0.03
MB 2% OTES, 2% in PE	17.98 ± 1.10	121.78 ± 0.56

* Values determined based on temperature sweep test; the applied angular frequency was 10 rad·s^−1^, and the applied oscillation strain was 0.02%.

**Table 10:** Calculated areas under the viscosity plots and the areas of viscosity loops.

	Area (A) Pas (1/s)	Area (B) Pas (1/s)	Area of Hysteresis Pas (1/s)
Polyethylene PE50	4256.79	4114.19	142.6
MB	3342.52	2877.64	464.88
MB_1% IBTMS	5786.45	3918.31	1868.14
MB_2% IBTMS	5184.78	3560.77	1624.01
MB_1%OTES	5032.27	2457.83	2574.44
MB_2% OTES	5889.59	3959.75	1929.84

Index A in the table means that the area was calculated under the viscosity graph measured using an increasing shear rate; index B means that the area was calculated under the viscosity graph that was measured using a decreasing shear rate.

**Table 11:** Calculated values of μ_0_ as zero shear viscosity, μ_∞_ as infinite viscosity, k as consistency, and n as the power law index and a parameter describing the transition between a Newtonian plateau and the power law region.

	μ_0_ (Pas)	μ_∞_ (Pas)	k (s)	n (-)	a (-)
MB, 2% in PE *	234.943	9.26208 × 10^−5^	8.63539 × 10^−3^	−15.5871	---
MB_1% IBTMS, 2% in PE	305.924	8.49663	2.16299 × 10^−3^	−4.38369	0.737365
MB_2% IBTMS, 2% in PE	253.487	0.998161	2.14472 × 10^−2^	−1.95371	1.49539
MB_1% OTES, 2% in PE	293.376	1.81277	1.88146 × 10^−2^	−3.25372	1.33434
MB_2% OTES, 2% in PE	273.310	0.867537	1.746997 × 10^−2^	−3.72423	1.63849

*—The viscosity data were fitted to the Carreau model.

**Table 12:** The temperatures at 5, 10, 20 weight loss T_5%_, T_10%_, T_20%_ and at the maximum of the DTA peak T_max_.

	T_1%_ (°C)	T_5%_ (°C)	T_10%_ (°C)	T_20%_ (°C)	T_max_ (°C)
PE	328	391	412	430	482.54
MB, 2% in PE	310	352	370	403	465.61
MB_1% IBTMS, 2% in PE	300	342	361	384	452.71
MB_2% IBTMS, 2% in PE	314	361	389	412	464.77
MB_1% OTES, 2% in PE	314	347	366	384	456.98
MB_2% OTES, 2% in PE	310	384	403	417	470.15

**Table 13:** The crystallization T_c_ and melting T_m_ temperatures, the values of crystallization enthalpy ΔH_c_ and melting enthalpy ΔH_m_, and the degree of crystallinity χ_c_.

	Tc (°C)	ΔHc (J·g−1)	Tm *(°C)	ΔHm *(J·g−1)	χc (%)
polyethylene	107.2	85.3	122.1	−71.97	24.56
MB, 2% in PE	114.0	93.33	124.1	−97.4	33.36
MB_1% IBTMS, 2% in PE	113.4	95.19	123.9	−92.39	31.65
MB_2% IBTMS, 2% in PE	113.6	118.5	123.8	−105.36	36.09
MB_1% OTES, 2% in PE	113.5	86.3	123.7	−81.54	27.93
MB_2% OTES, 2% in PE	113.5	99.33	123.7	−98.00	33.57

* The values of enthalpy of melting and temperatures of melting were determined for a second heating of the sample after removing the thermal history of the sample.

## Data Availability

The original contributions presented in the study are included in the article/Supplementary Materials, further inquiries can be directed to the corresponding author.

## Supplementary Materials

The following supporting information can be downloaded at: https://www.mdpi.com/article/10.3390/polym17050618/s1, Figure S1. The DLS plot. The size of the pigment RED 122 and aggregates modified by isobutyltrimethoxysilane IBTMS pigment formed in water medium; Figure S2. The DLS plot. The size of the pigment RED 122 and aggregates modified by octyltriethoxysilane OTES pigment formed in water medium.; Figure S3. The DLS plot. The size of the pigment RED 122 and aggregates modified by isobutyltrimethoxysilane IBTMS pigment formed in paraffine oil medium; Figure S4. The DLS plot. The size of the pigment RED 122 and aggregates modified by octyltriethoxysilane OTES pigment formed in paraffine oil medium; Figure S5. The SEM pictures of pigment RED 122 modified with 2 wt.% of isobutylotrimetoxysilane IBTMS (SEM microscope, LEO 1530 Gemini, producent Zeiss/Leo, Oberkochen, Germany); Figure S6. The SEM pictures of pigment RED 122 modified with 1 wt% of octylotriethoxysilane OTES (SEM microscope, LEO 1530 Gemini, producent Zeiss/Leo, Oberkochen, Germany); Figure S7. The SEM pictures of pigment RED 122 modified with 2 wt% of octylotriethoxysilane OTES (SEM microscope, LEO 1530 Gemini, producent Zeiss/Leo, Oberkochen, Germany); Figure S8. Reflectance plot for the RED 122 pigment modified with 2 wt.% of isobutyltrimethoxysilane IBTMS; Figure S9. Reflectance plot for the RED 122 pigment modified with 2 wt.% of octyltriethoxysilane OTES; Figure S10. Storage shear modulus G′ and loss shear modulus G″ measured at 170 °C as a function of oscillation strain up to 5% (applied angular frequency 10 rad·s^−1^) and angular frequency (applied oscillation strain 0.5%) for polyethylene containing 2 wt.% of various color concentrates. Figure S11. Storage shear modulus G′ and loss shear modulus G″ as a function of temperature for color concentrates containing pigment RED 122 and silane-modified pigments. The applied oscillation strain 0.02%, the applied angular frequency 10 rad·s^−1^; Figure S12. Storage shear modulus G′ and loss shear modulus G″ measured as a function of temperature for polyethylene containing 2 wt.% of various colored masterbatches, applied oscillation strain 0.02%, the applied angular frequency 10 rad·s^−1^; Figure S13. Shear viscosity μ of masterbatches containing RED 122 pigment and polyethylene measured at 170 °C as a function of shear rate. The index A in the graph means that the viscosity was measured using an increasing shear rate, the index B means that the viscosity was measured using a decreasing shear rate; Figure S14. Shear viscosity μ of polyethylene containing 2 wt.% of various color concentrates. The rotational tests were carried out at 170 °C as a function of applied shear rate in range 0.001–300 s^−1^; Figure S15. TGA plots for polyethylene containing 2 wt.% of various color concentrates. Figure S16. DSC plots for polyethylene used as a pigment carrier in color concentrates; Figure S17. DSC plots for polyethylene containing 2% wt. of color concentrate containing pigment RED 122 modified by 2 wt.% of OTES (octadecyltriethoxysilane); Figure S18. DSC plots for polyethylene containing 2% wt. of color concentrate containing pigment RED 122 modified by 2 wt.% of IBTMS (isobutylotrimethoxysilane).

## References

[1] Witucki G.L. (1993). Silane Primer: Chemistry and Applications of AIkoxy Silanes. J. Coat. Technol..

[2] Deschler U., Kleinschmit P., Panster P. (1986). 3-Chloropropyltrialkoxy Silanes—Key Intermediates for the Commercial Production of Organofunctionalized Silanes and Polysiloxanes. Angew. Chem. Int. Ed..

[3] Zhu D., Hu N., Schaefer D.W., Zarras P., Soucek M.D., Atul Tiwari A. (2020). Water-based sol–gel coatings for military coating applications, chapter 1. Handbook of Waterborne Coatings.

[4] Wang D., Ren F., Zhu C., Feng J., Cheng Q., Chen S., Shen G., Wang F. (2019). Hybrid silane technology in silica-reinforced tread compound. Rubber Chem. Technol..

[5] Máková V., Holubová B., Krabicová I., Kulhánková J., Řezanka M. (2021). Hybrid organosilane fibrous materials and their contribution to modern science. Polymer.

[6] Antonucci J.M., Dickens S.H., Fowler B.O., Xu H. (2005). Chemistry of Silanes-Interfaces in Dental Polymers and Composites. J. Res. Natl. Inst. Stand. Technol..

[7] Figueira R.B. (2020). Hybrid sol-gel coatings for corrosion mitigation: A critical review. Polymers.

[8] Bouibed A., Doufnoune R. (2019). Synthesis and characterization of hybrid materials based on graphene oxide and silica nanoparticles and their effect on the corrosion protection properties of epoxy resin coatings. J. Adhes. Sci. Technol..

[9] Yamazaki R., Karyu N., Noda M., Fujii S., Nakamura Y. (2016). Quantitative measurement of physisorbed silane on a silica particle surface treated with silane coupling agents by thermogravimetric analysis. J. Appl. Polym. Sci..

[10] Sanchez C., Julián B., Belleville P., Popall M. (2005). Applications of hybrid organic-inorganic nanocomposites. J. Mater. Chem..

[11] Matinlinna J.P., Vallittu P.K., Lassila L.V.A. (2011). Effects of different silane coupling agent monomers on flexural strength of an experimental filled resin composite. J. Adhes. Sci. Technol..

[12] Abdulhameed J.I., Ali A.H., Kara I.H. (2022). Developing Crumbed Rubber Tires/Epoxy Composite, by Surface Treatment with Different Silane Coupling Agents. Mater. Sci. Forum.

[13] Pokorný P., Kouřil M. (2024). Predicted Corrosion Performance of Organofunctional Silane Coated Steel Reinforcement for Concrete Structures: An Overview. Buildings.

[14] Lung C.Y.K., Matinlinna J.P. (2012). Aspects of silane coupling agents and surface conditioning in dentistry: An overview. Dent. Mater..

[15] Thetford D., Cherryman J., Chorlton A.P., Docherty R. (2004). Theoretical molecular modelling calculations on the solid state structure of some organic pigments. Dye. Pigment..

[16] Saito Y., Iwamoto S., Hontama N., Tanaka Y., Endo T. (2020). Dispersion of quinacridone pigments using cellulose nanofibers promoted by CH–π interactions and hydrogen bonds. Cellulose.

[17] Rossin E., Yang Y., Chirico M., Rossi G., Galloni P., Sartorel A. (2024). Quinacridone dyes: Versatile molecules and materials for photo- and photoelectrochemical processes. Energy Adv..

[18] Broda J., Dogan F. (2012). Structure of Polypropylene Fibres Coloured with Organic Pigments. Chapter 25. Polypropylene.

[19] Kanbur Y., Coskun H., Głowacki E.D., Irimia-Vladu M., Sariciftci N.S., Yumusak C. (2019). High temperature-stability of organic thin-film transistors based on quinacridone pigments. Org. Electron..

[20] Głowacki E.D., Leonat L., Irimia-Vladu M., Schwödiauer R., Ullah M., Sitter H., Bauer S., Sariciftci N.S. (2012). Intermolecular hydrogen-bonded organic semiconductors-Quinacridone versus pentacene. Appl. Phys. Lett..

[21] Kim D.H., Jeon S.J., Han Y.W., Kim Y.H., Yang N.G., Lee H.S., Moon D.K. (2021). Design and synthesis of the quinacridone-based donor polymers for application to organic solar cells. J. Ind. Eng. Chem..

[22] Hao Z., Iqbal A. (1997). Some aspects of organic pigments. Chem. Soc. Rev..

[23] Zeng H., Fan X., He X., Jiang Z., Niu L., Yuan H. (2025). Influence of anchoring group of dispersants on the dispersion performance of Pigment Red 122. Dye. Pigment..

[24] Agbo C., Acheampong C., Zhang L., Li M., Wang D., Fu S. (2019). Synthesis and application of novel dispersant using a dichlorotriazine azo moiety and Dodecan-1-ol. Prog. Org. Coat..

[25] Tsubokawa N., Kobayashi M., Ogasawara T. (1999). Graft polymerization of vinyl monomers initiated by azo groups introduced onto organic pigment surface. Prog. Org. Coat..

[26] Elgammal M., Schneider R., Gradzielski M. (2016). Development of self-curable hybrid pigment inks by miniemulsion polymerization for inkjet printing of cotton fabrics. Dye. Pigment..

[27] Ding Y., Ye M., Han A., Zang Y. (2018). Preparation and characterization of encapsulated C.I. pigment yellow 12 via ball-milling and mini-emulsion polymerization. Prog. Org. Coat..

[28] Saito Y., Iwamoto S., Tanaka Y., Hontama N., Endo T. (2021). Suppressing aggregation of quinacridone pigment and improving its color strength by using chitosan nanofibers. Carbohydr. Polym..

[29] Marszalek T., Krygier I., Pron A., Wrobel Z., Blom P.M.W., Kulszewicz-Bajer I., Pisula W. (2019). Self-assembly and charge carrier transport of sublimated dialkyl substituted quinacridones. Org. Electron..

[30] Thambiliyagodage C., Wijesekera R. (2022). Ball milling—A green and sustainable technique for the preparation of titanium based materials from ilmenite. Curr. Res. Green Sustain. Chem..

[31] Boudria A., Hammoui Y., Adjeroud N., Djerrada N., Madani K. (2018). Effect of filler load and high-energy ball milling process on properties of plasticized wheat gluten/olive pomace biocomposite. Adv. Powder Technol..

[32] Loh Z.H., Samanta A.K., Heng P.W.S. (2015). Overview of milling techniques for improving the solubility of poorly water-soluble drugs. Asian J. Sci..

[33] Piras C.C., Fernández-Prieto S., De Borggraeve W.M. (2019). Ball milling: A green technology for the preparation and functionalisation of nanocellulose derivatives. Nanoscale Adv..

[34] Ferreto H.F.R., Oliveira A.C.F., Lima L.F.C.P., Parra D.F., Lugão A.B. (2012). Thermal, tensile and rheological properties of linear low density polyethylene (LLDPE) irradiated by gamma-ray in different atmospheres. Radiat. Phys. Chem..

[35] Xu Y., Zhou R., Mu J., Ding Y., Jiang J. (2022). Synergistic flame retardancy of linear low-density polyethylene with surface modified intumescent flame retardant and zinc borate. Colloids Surf. A Physicochem. Eng. Asp..

[36] Salakhov I.I., Shaidullin N.M., Chalykh A.E., Matsko M.A., Shapagin A.V., Batyrshin A.Z., Shandryuk G.A., Nifant’ev I.E. (2021). Low-Temperature Mechanical Properties of High-Density and Low-Density Polyethylene and Their Blends. Polymers.

[37] Chorobiński M., Skowroński Ł., Bieliński M. (2019). Methodology for determining selected characteristics of polyethylene dyeing using CIELab system. Polimery.

[38] Neo P.K., Kitada Y., Deeying J., Thumsorn S., Soon M.F., Goh Q.S., Leong Y.W., Ito H. (2023). Influence of Compounding Parameters on Color Space and Properties of Thermoplastics with Ultramarine Blue Pigment. Polymers.

[39] Jin S.B., Hao L.T., Hwang S.Y., Oh D.X., Koo J.M., Jeon H., Park S.B., Park J. (2022). Masterbatch of Chitosan Nanowhiskers for Preparation of Nylon 6,10 Nanocomposite by Melt Blending. Polymers.

[40] Ibáñez-García A., Berbegal-Pina R., Vidal R., Martínez-García A. (2024). Sustainability in the Development of Natural Pigment-Based Colour Masterbatches and Their Application in Biopolymers. Polymers.

[41] Ullah J., Harkin-Jones E., Mcllhagger A., Magee C., Tormey D., Dave F., Sherlock R., Dixon D. (2022). The effect of masterbatch pigments on the crystallization, morphology, and shrinkage behaviour of isotactic polypropylene. J. Polym. Res..

[42] Wu S., Brzozowski K.J. (1971). Surface free energy and polarity of organic pigments. J. Coll. Interface Sci..

[43] Li J., Touil I., de Alba C.F., Boisson F., Boyron O., Narimissa E., Lu B., Zhang H., Maazouz A., Lamnawar K. (2023). Structure-rheology properties of polyethylenes with varying macromolecular architectures. J. Polym. Res..

[44] Agrawal P., Silva M.H.A., Cavalcanti S.N., Freitas D.M.G., Araújo J.P., Oliveira A.D.B., Mélo T.J.A. (2022). Rheological properties of high-density polyethylene/linear low-density polyethylene and high-density polyethylene/low-density polyethylene blends. Polym. Bull..

[45] TA Instruments Understanding the Rheology of Thermoplastic Polymers.

[46] Gurt A., Khonsari M. (2024). A review of the rheological consistency of materials. Lubricants.

[47] (2013). Standard Test Method for Melt Flow Rates of Thermoplastics by Extrusion Plastometer.

[48] (2018). Standard Test Method for Density of Plastics by the Density-Gradient Technique.

[49] Kaelble D.H. (1970). Dispersion-polar surface tension properties of organic solids. J. Adhes..

[50] Molenda M., Wrona E., Siwiec J. (2012). Zastosowanie modelu CIELab w badaniach barwy lotnych popiołów. Probl. Eksploat..

[51] Li D., Zhou L., Wang X., He L., Yang X. (2019). Effect of Crystallinity of Polyethylene with Different Densities on Breakdown Strength and Conductance Property. Materials.

[52] Alapati S., Meledath J.T., Karmarkar A. (2014). Effect of Morphology on Electrical Treeing in Low Density Polyethylene Nanocomposites. IET Sci. Meas. Technol..

[53] Niedermeier W., Fröhlich J., Luginsland H.-D. (2002). Reinforcement mechanism in the rubber matrix by active fillers. Kaut. Gummi Kunstst..

[54] Sarkawi S.S., Dierkes W.K., Noordermeer J.W.M. (2014). Effect of a Silane Coupling Agent on the Morphology of Silica Reinforced Natural Rubber. Kaut. Gummi Kunstst..

[55] Payne A.R. (1962). The dynamic properties of carbon black-loaded natural rubber vulcanizates Part I. J. Appl. Polym. Sci..

[56] Zhong X., Song Y., Zheng Q. (2023). Payne effect and Mullins effect of silica filled butadiene rubber nanocomposites vulcanizates and their unextractable gels. Polymer.

[57] Malkin A.Y. (2006). Continuous relaxation spectrum—Its advantages and methods of calculation. Int. J. Appl. Mech. Eng..

[58] Bartenev G.M., Valishin A.A., Panchuk I.I. (1977). Relaxation spectrometry of elastomers. Vysokomol. Soyed..

[59] Malkin A.Y., Vasilyev G.B., Adrianov A.V. (2010). On continuous relaxation spectrum. Method of calculation. Polym. Sci. Ser. A.

[60] Wilczyński K., Ładna E. (2001). Modele reologiczne cieczy lepkich. Reologia w Przetwórstwie Tworzyw Sztucznych.

