# Supplementary material for: Rheological Properties of Polyethylene Color Masterbatches Containing Pigment RED 122 (2,9-Dimethylquinacridone) Modified by Silanes Using Pulverization Method
Source: Polymers (Basel). 2025 Feb 25;17(5):618. doi: 10.3390/polym17050618 (PMC11902374; doi:10.3390/polym17050618)
Supplement: Supplementary file 1 [file polymers-17-00618-s001.zip › polymers-3484651-supplementary.pdf]

Rheological Properties of Polyethylene Color Masterbatches Containing Pigment Red 122 (2,9-dimethylquinacridone) Modified by Silanes Using Pulverization Method

Magdalena Kozłowska, Magdalena Lipińska * and Michał Okraska

Institute of Polymer and Dye Technology, Lodz University of Technology, 90-537 Lodz, Poland; magdalena.stefaniak@dokt.p.lodz.pl (M.K.); michal.okraska@p.lodz.pl (M.O.)
* Correspondence: magdalena.lipinska@p.lodz.pl

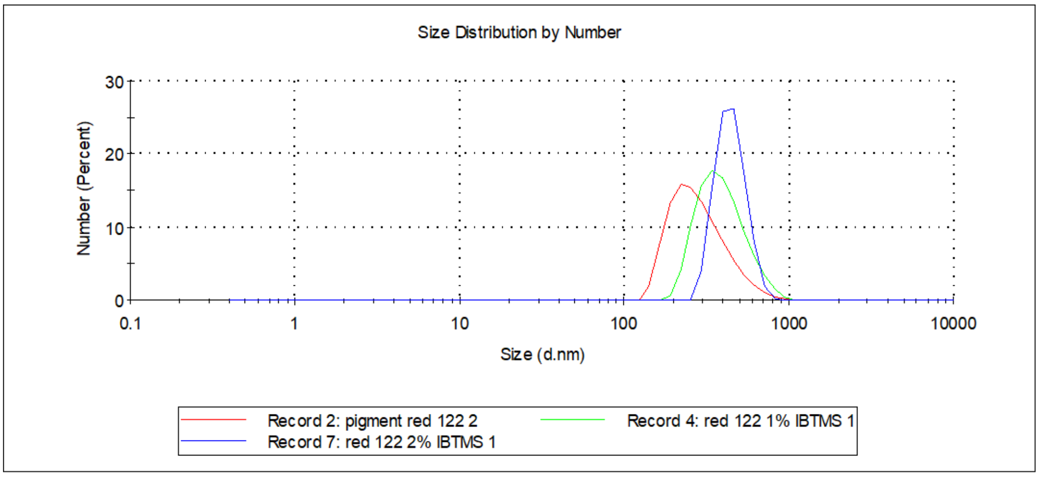

Figure S1. The DLS plot. The size of the pigment RED 122 and modified by isobutyltrimethoxysilane IBTMS pigment aggregates formed in water medium.

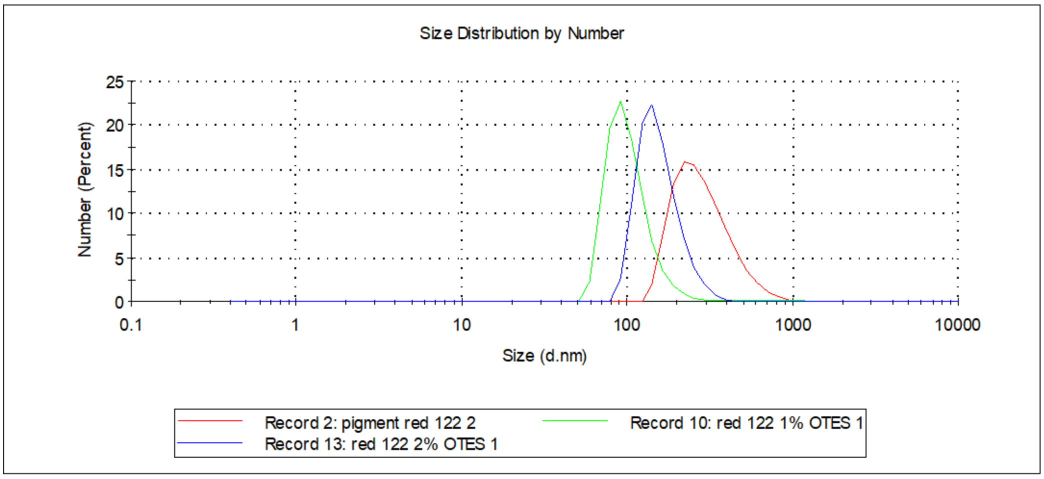

Figure S2. The DLS plot. The size of the pigment RED 122 and modified by octyltriethoxysilane OTES pigment aggregates formed in water medium.

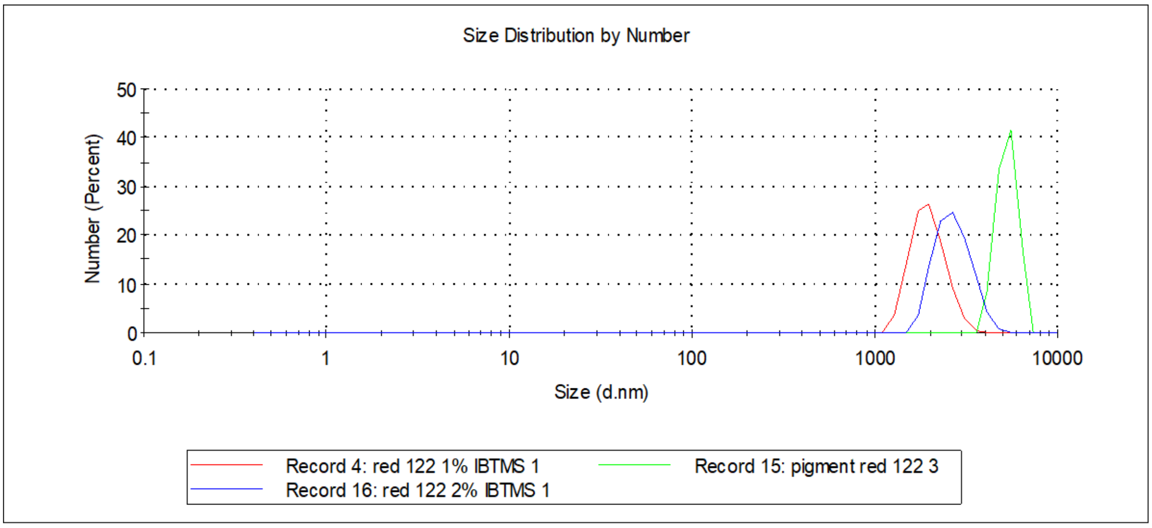

Figure S3. The DLS plot. The size of the pigment RED 122 and modified by isobutyltrimethoxysilane IBTMS pigment aggregates formed in paraffine oil medium.

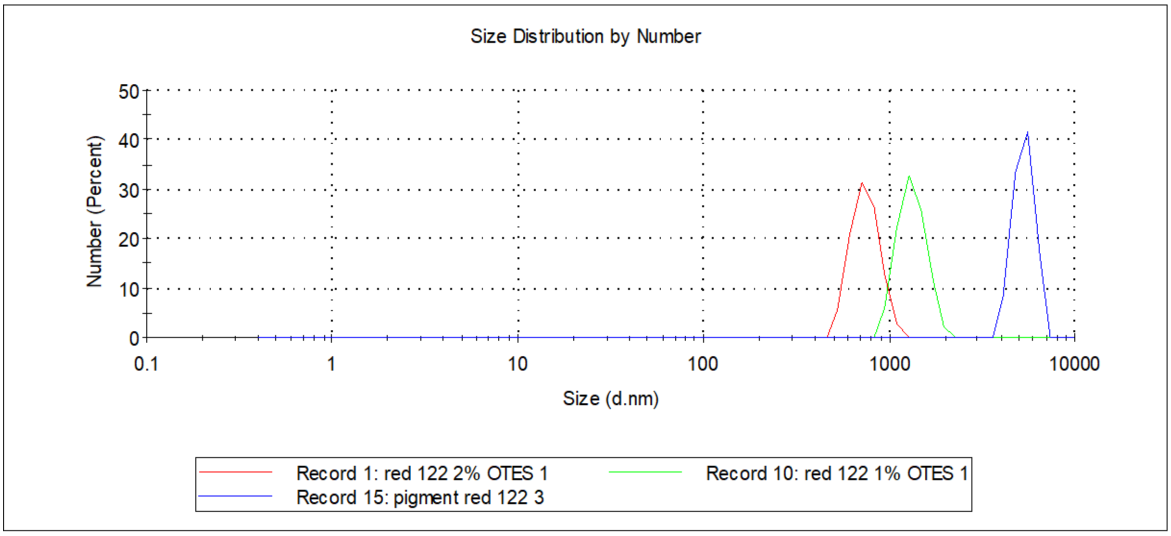

Figure S4. The DLS plot. The size of the pigment RED 122 and modified by octyltriethoxysilane OTES pigment aggregates formed in paraffine oil medium.

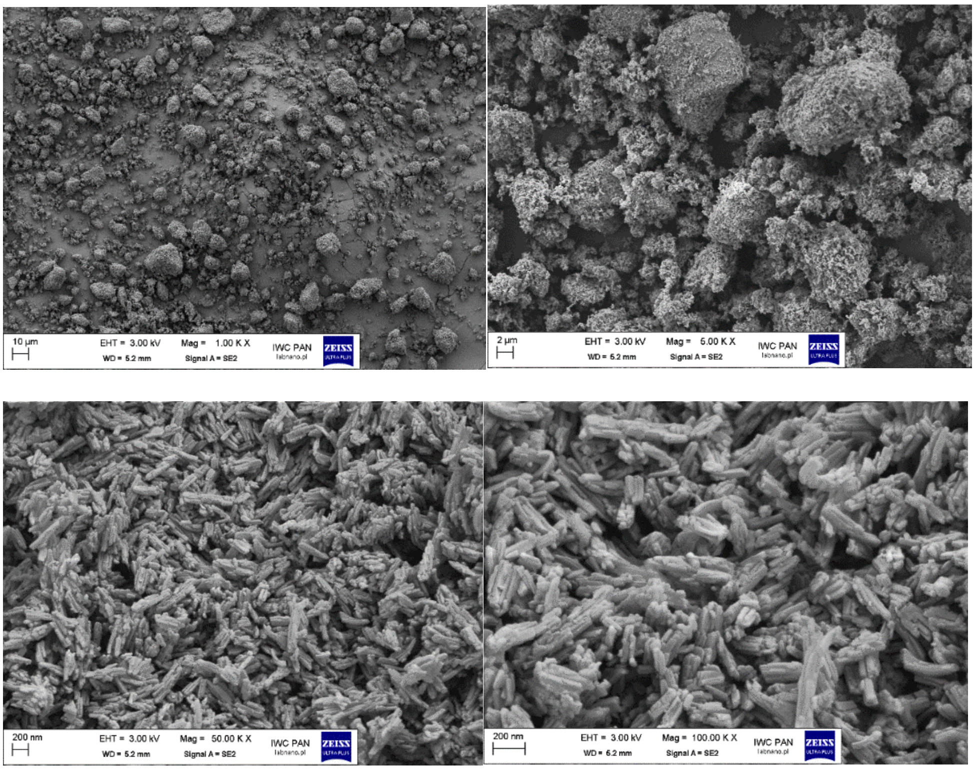

Figure S5. The SEM pictures of pigment RED 122 modified with 2 wt% of isobutylotrimetoxysilane IBTMS (SEM microscope, LEO 1530 Gemini, producent Zeiss/Leo, Oberkochen, Germany).

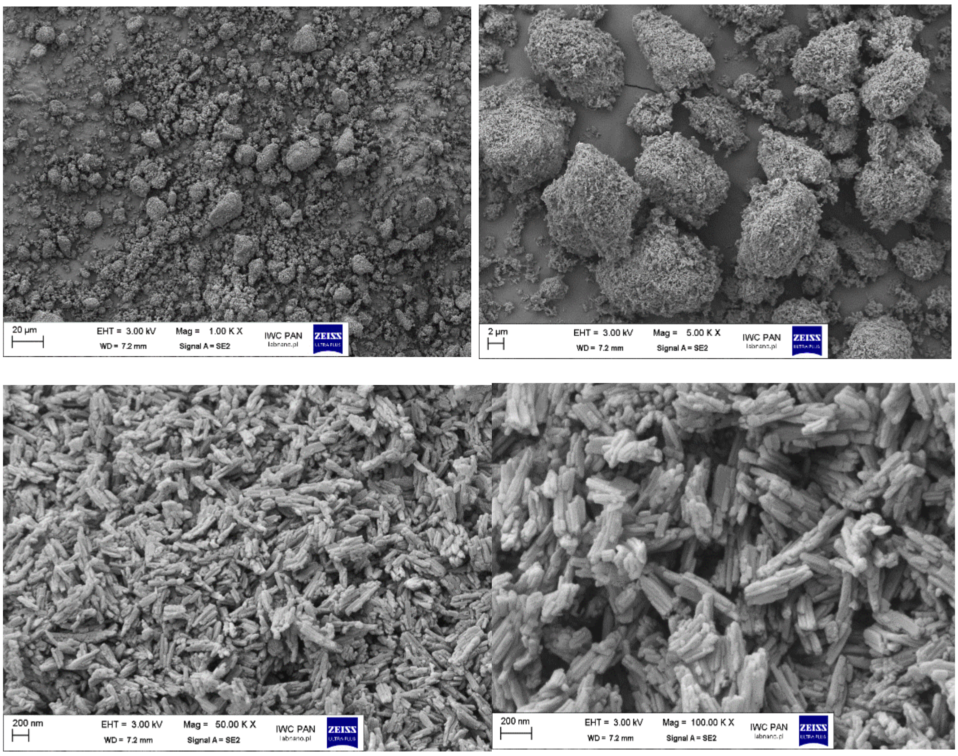

Figure S6. The SEM pictures of pigment RED 122 modified with 1 wt% of octylotriethoxysilane OTES (SEM microscope, LEO 1530 Gemini, producent Zeiss/Leo, Oberkochen, Germany).

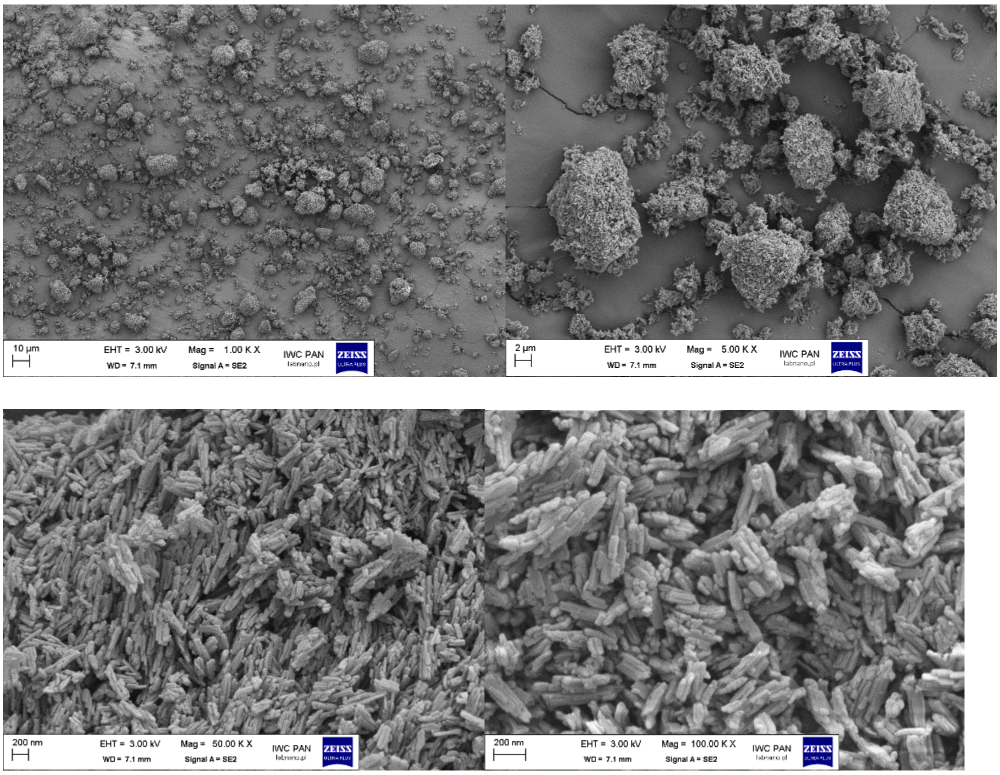

Figure S7. The SEM pictures of pigment RED 122 modified with 2 wt% of octylotriethoxysilane OTES (SEM microscope, LEO 1530 Gemini, producent Zeiss/Leo, Oberkochen, Germany).

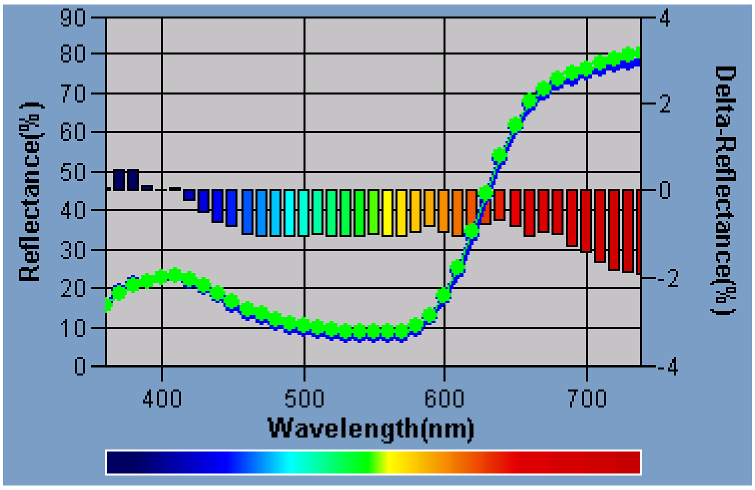

Figure S8. Reflectance plot for the RED 122 pigment modified with 2 wt.% of isobutyltrimethoxysilane IBTMS.

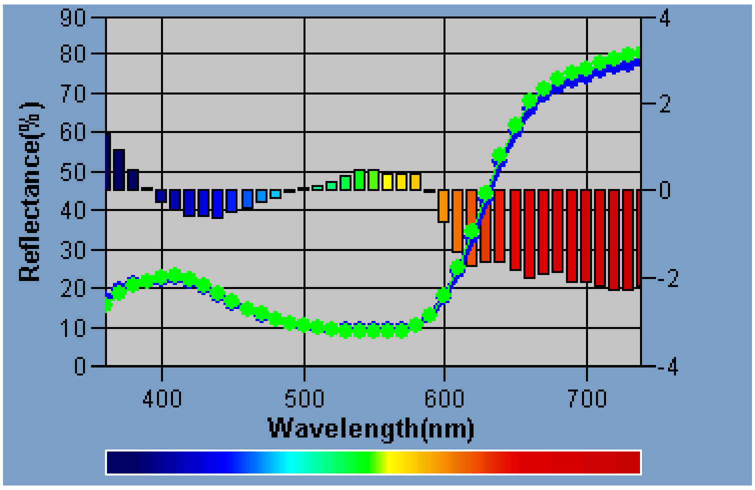

Figure S9. Reflectance plot for the RED 122 pigment modified with 2 wt.% of octyltriethoxysilane OTES.

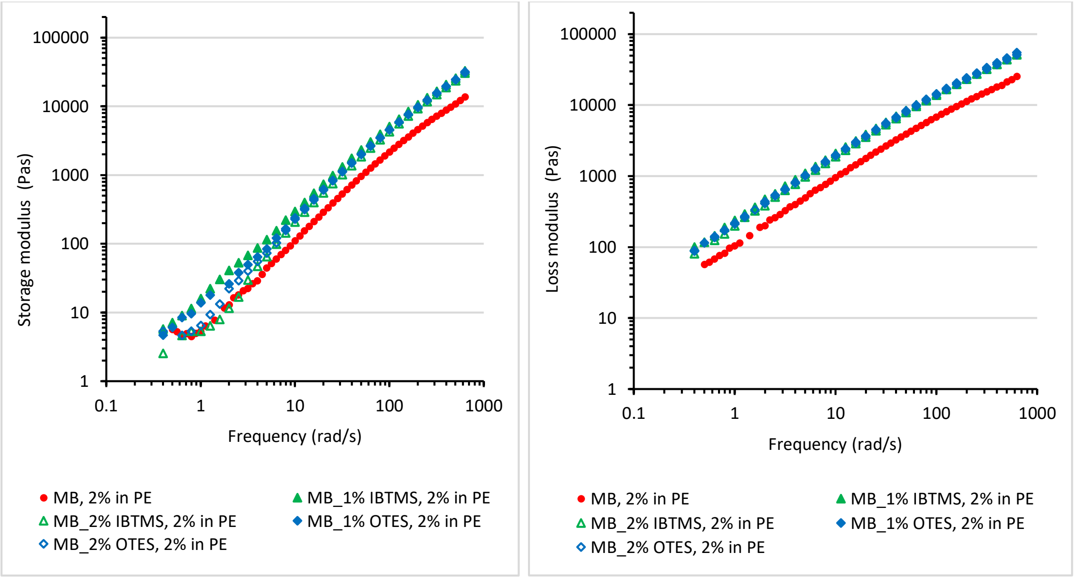

Figure S10. Storage shear modulus G′ and loss shear modulus G′′ measured at 170°C as a function of angular frequency for polyethylene containing 2 wt.% of various color concentrates. The applied oscillation strain 0.5%.

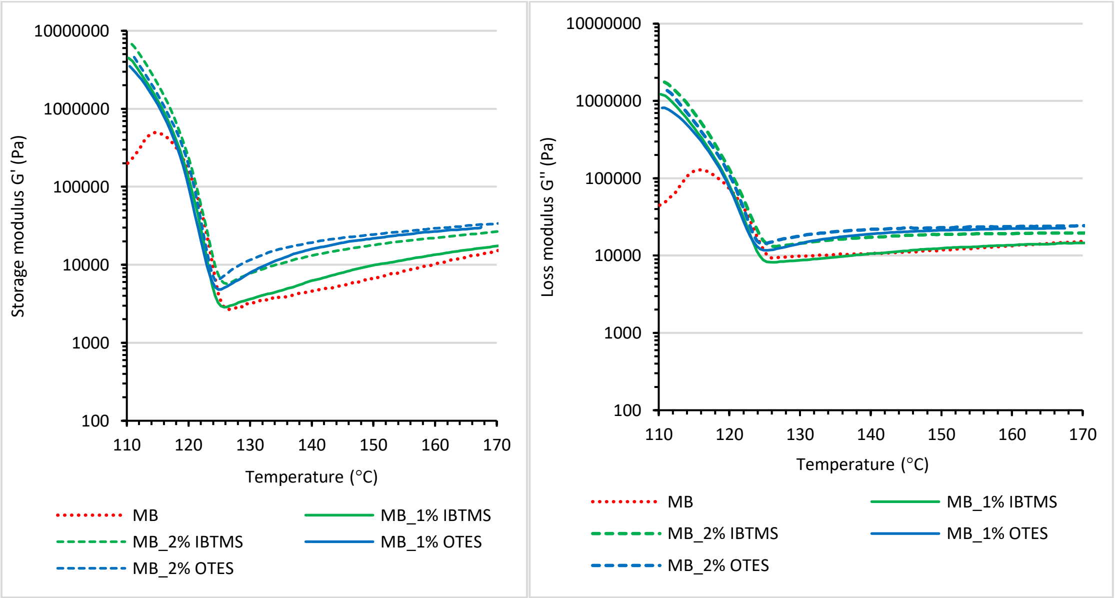

Figure S11. Storage shear modulus G′ and loss shear modulus G′′ as a function of temperature for color concentrates containing pigment RED 122 and silane modified pigments. The applied oscillation strain 0.02%, the applied angular frequency 10 rad·s^{-1}.

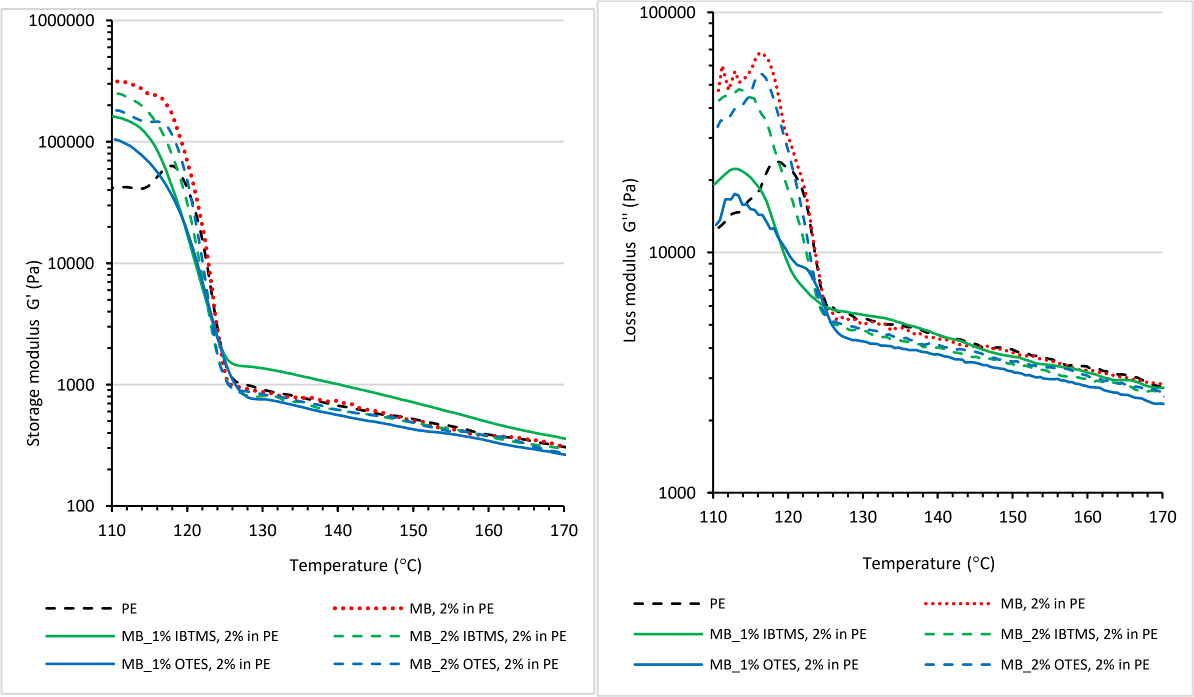

Figure S12. Storage shear modulus G′ and loss shear modulus G′′ measured as a function of temperature for polyethylene containing 2 wt.% of various colored masterbatches, applied oscillation strain 0.02%, the applied angular frequency 10 rad·s^{-1}.

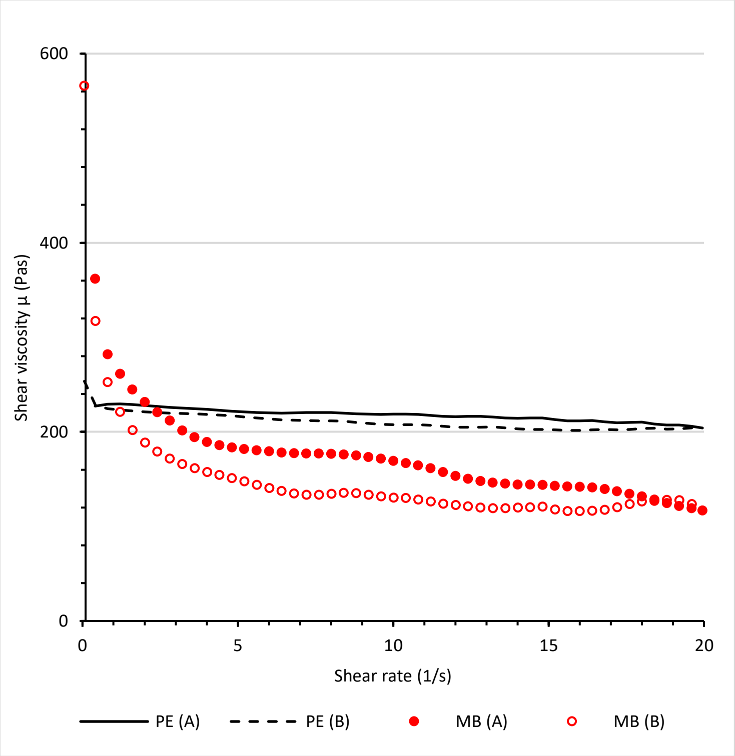

Figure S13. Shear viscosity μ of masterbatches containing RED 122 pigment and polyethylene measured at 170°C as a function of shear rate. The index A in the graph means that the viscosity was measured using an increasing shear rate, the index B means that the viscosity was measured using a decreasing shear rate.

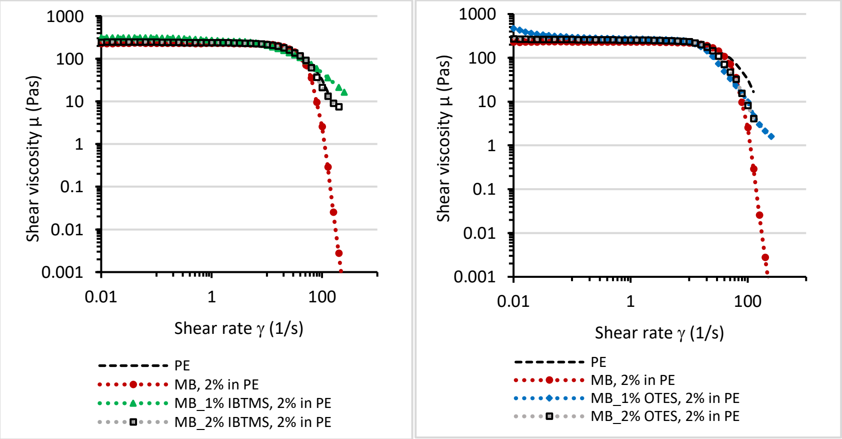

Figure S14. Shear viscosity μ of polyethylene containing 2 wt.% of various color concentrates. The rotational tests were done at 170°C as a function of applied shear rate in range 0.001 – 300 s^{-1}.

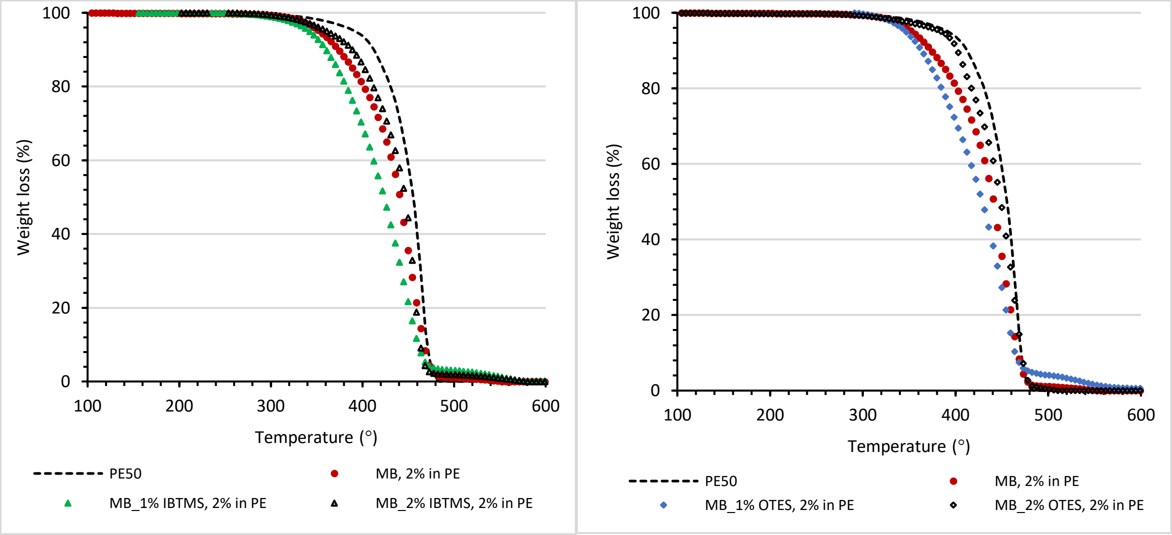

Figure S15. TGA plots for polyethylene containing 2 wt.% of various color concentrates.

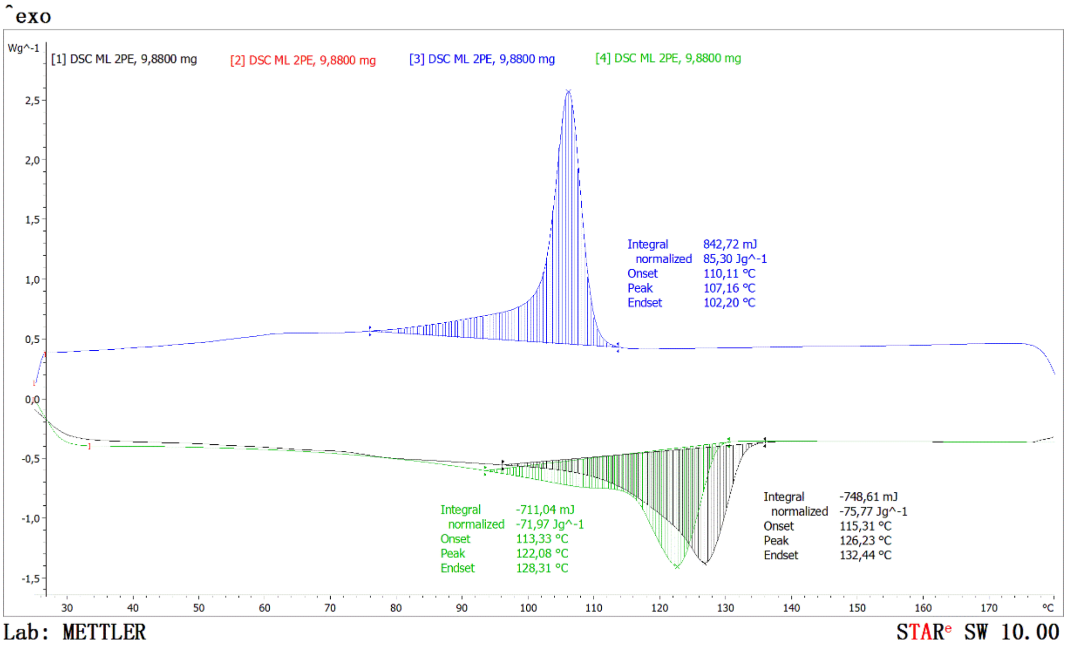

Figure S16. DSC plots for polyethylene used as a pigment carrier in color concentrates.

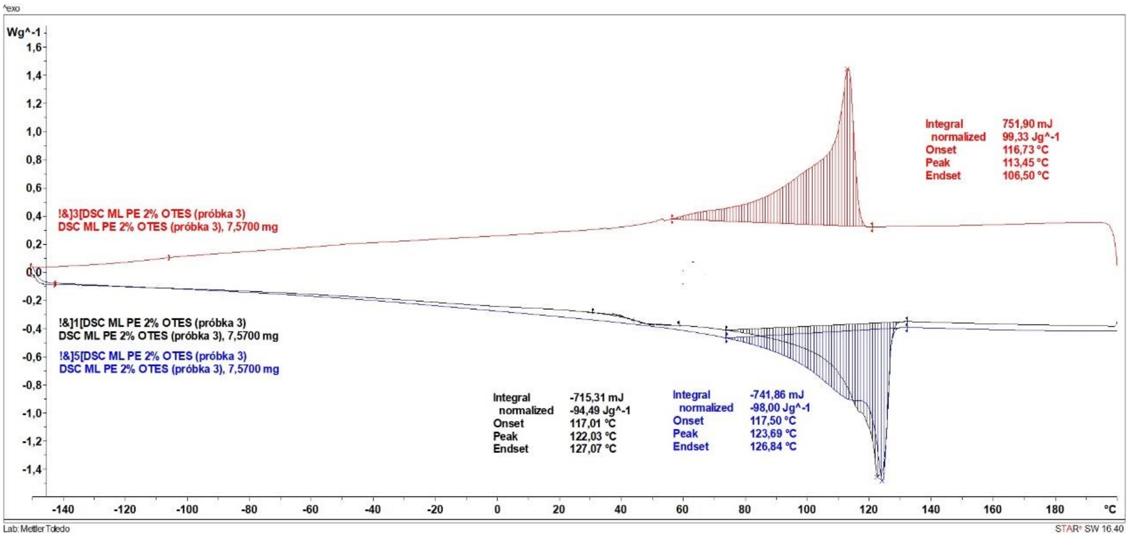

Figure S17. DSC plots for polyethylene containing 2% wt. of color concentrate containing pigment RED 122 modified by 2 wt.% of OTES (octadecyltriethoxysilane).

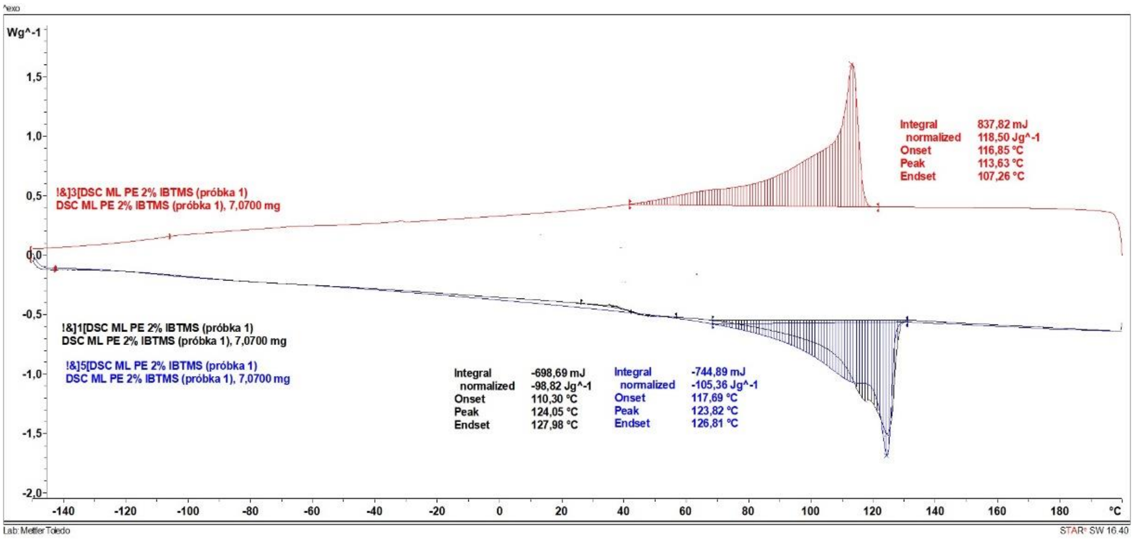

Figure S18. DSC plots for polyethylene containing 2% wt. of color concentrate containing pigment RED 122 modified by 2 wt.% of IBTMS (isobutylotrimethoxysilane).
